# Association of adverse perinatal outcomes of intrahepatic cholestasis of pregnancy with biochemical markers: results of aggregate and individual patient data meta-analyses

**DOI:** 10.1016/S0140-6736(18)31877-4

**Published:** 2019-03-02

**Authors:** Caroline Ovadia, Paul T Seed, Alexandros Sklavounos, Victoria Geenes, Chiara Di Illio, Jenny Chambers, Katherine Kohari, Yannick Bacq, Nuray Bozkurt, Romana Brun-Furrer, Laura Bull, Maria C Estiú, Monika Grymowicz, Berrin Gunaydin, William M Hague, Christian Haslinger, Yayi Hu, Tetsuya Kawakita, Ayse G Kebapcilar, Levent Kebapcilar, Jūratė Kondrackienė, Maria P H Koster, Aneta Kowalska-Kańka, Limas Kupčinskas, Richard H Lee, Anna Locatelli, Rocio I R Macias, Hanns-Ulrich Marschall, Martijn A Oudijk, Yael Raz, Eli Rimon, Dan Shan, Yong Shao, Rachel Tribe, Valeria Tripodi, Cigdem Yayla Abide, Ilter Yenidede, Jim G Thornton, Lucy C Chappell, Catherine Williamson

**Affiliations:** a Department of Women and Children's Health, King's College London, London, UK; b Women's Health Research Centre, Imperial College London, London, UK; c Department of Obstetrics, Gynecology and Reproductive Sciences, Yale School of Medicine, New Haven, CT, USA; d Department of Hepatology and Gastroenterology, University Hospital of Tours, Tours, France; e Department of Obstetrics and Gynecology, Gazi University School of Medicine, Ankara, Turkey; f Department of Anesthesiology and Reanimation, Gazi University School of Medicine, Ankara, Turkey; g Division of Obstetrics, University Hospital of Zurich, Zurich, Switzerland; h Department of Medicine and Institute for Human Genetics, University of California, San Francisco, CA, USA; i Ramón Sardá Mother's and Children's Hospital, Buenos Aires, Argentina; j Department of Gynecological Endocrinology, Warsaw Medical University, Warsaw, Poland; k Robinson Research Institute, University of Adelaide, Adelaide, SA, Australia; l Department of Obstetrics and Gynecology, West China Second University Hospital, Sichuan University, Chengdu, Sichuan, China; m Department of Obstetrics and Gynecology, MedStar Washington Hospital Center, Washington, DC, USA; n Department of Gynecology and Obstetrics, Selcuk University, Konya, Turkey; o Internal Medicine, Selcuk University, Konya, Turkey; p Department of Gastroenterology, Lithuanian University of Health Sciences, Kaunas, Lithuania; q Institute for Digestive Research, Lithuanian University of Health Sciences, Kaunas, Lithuania; r Department of Obstetrics and Gynaecology, Erasmus MC, Rotterdam, Netherlands; s Obstetrics and Gynaecology Clinic, Institute of Mother and Child, Warsaw, Poland; t Obstetrics and Gynecology, Keck School of Medicine University of Southern California, Los Angeles, CA, USA; u Department of Obstetrics and Gynecology, University of Milano-Bicocca, Monza, Italy; v National Institute for the Study of Liver and Gastrointestinal Diseases, Institute of Biomedical Research of Salamanca, University of Salamanca, Salamanca, Spain; w Department of Molecular and Clinical Medicine, University of Gothenburg, Gothenburg, Sweden; x Department of Obstetrics, Amsterdam University Medical Center, University of Amsterdam, Amsterdam, Netherlands; y Department of Obstetrics and Gynecology, Tel Aviv Medical Center, Sackler Faculty of Medicine, Tel Aviv, Israel; z Department of Obstetrics and Gynecology, The First Affiliated Hospital of Chongqing Medical University, Chongqing, China; aa School of Pharmacy and Biochemistry, University of Buenos Aires, Buenos Aires, Argentina; ab Clinic of Obstetrics and Gynecology, Zeynep Kamil Women and Children's Health Training and Research Hospital, University of Health Sciences, Istanbul, Turkey; ac Division of Child Health, Obstetrics and Gynaecology, University of Nottingham, Nottingham, UK

## Abstract

**Background:**

Intrahepatic cholestasis of pregnancy is associated with adverse perinatal outcomes, but the association with the concentration of specific biochemical markers is unclear. We aimed to quantify the adverse perinatal effects of intrahepatic cholestasis of pregnancy in women with increased serum bile acid concentrations and determine whether elevated bile acid concentrations were associated with the risk of stillbirth and preterm birth.

**Methods:**

We did a systematic review by searching PubMed, Web of Science, and Embase databases for studies published from database inception to June 1, 2018, reporting perinatal outcomes for women with intrahepatic cholestasis of pregnancy when serum bile acid concentrations were available. Inclusion criteria were studies defining intrahepatic cholestasis of pregnancy based upon pruritus and elevated serum bile acid concentrations, with or without raised liver aminotransferase concentrations. Eligible studies were case-control, cohort, and population-based studies, and randomised controlled trials, with at least 30 participants, and that reported bile acid concentrations and perinatal outcomes. Studies at potential higher risk of reporter bias were excluded, including case reports, studies not comprising cohorts, or successive cases seen in a unit; we also excluded studies with high risk of bias from groups selected (eg, a subgroup of babies with poor outcomes were explicitly excluded), conference abstracts, and Letters to the Editor without clear peer review. We also included unpublished data from two UK hospitals. We did a random effects meta-analysis to determine risk of adverse perinatal outcomes. Aggregate data for maternal and perinatal outcomes were extracted from case-control studies, and individual patient data (IPD) were requested from study authors for all types of study (as no control group was required for the IPD analysis) to assess associations between biochemical markers and adverse outcomes using logistic and stepwise logistic regression. This study is registered with PROSPERO, number CRD42017069134.

**Findings:**

We assessed 109 full-text articles, of which 23 studies were eligible for the aggregate data meta-analysis (5557 intrahepatic cholestasis of pregnancy cases and 165 136 controls), and 27 provided IPD (5269 intrahepatic cholestasis of pregnancy cases). Stillbirth occurred in 45 (0·83%) of 4936 intrahepatic cholestasis of pregnancy cases and 519 (0·32%) of 163 947 control pregnancies (odds ratio [OR] 1·46 [95% CI 0·73–2·89]; *I*^2^=59·8%). In singleton pregnancies, stillbirth was associated with maximum total bile acid concentration (area under the receiver operating characteristic curve [ROC AUC]) 0·83 [95% CI 0·74–0·92]), but not alanine aminotransferase (ROC AUC 0·46 [0·35–0·57]). For singleton pregnancies, the prevalence of stillbirth was three (0·13%; 95% CI 0·02–0·38) of 2310 intrahepatic cholestasis of pregnancy cases in women with serum total bile acids of less than 40 μmol/L versus four (0·28%; 0·08–0·72) of 1412 cases with total bile acids of 40–99 μmol/L (hazard ratio [HR] 2·35 [95% CI 0·52–10·50]; p=0·26), and versus 18 (3·44%; 2·05–5·37) of 524 cases for bile acids of 100 μmol/L or more (HR 30·50 [8·83–105·30]; p<0·0001).

**Interpretation:**

The risk of stillbirth is increased in women with intrahepatic cholestasis of pregnancy and singleton pregnancies when serum bile acids concentrations are of 100 μmol/L or more. Because most women with intrahepatic cholestasis of pregnancy have bile acids below this concentration, they can probably be reassured that the risk of stillbirth is similar to that of pregnant women in the general population, provided repeat bile acid testing is done until delivery.

**Funding:**

Tommy's, ICP Support, UK National Institute of Health Research, Wellcome Trust, and Genesis Research Trust.

**Research in context**

**Evidence before this study**

Intrahepatic cholestasis of pregnancy has been associated with increased risks of preterm birth and possibly stillbirth in cohort and population studies, yet no consensus has been reached within the medical community regarding the magnitude of its detrimental effects. Particularly, the adverse long-term effects of preterm birth are of relevance to many health-care professionals besides those delivering maternity care. Evidence from clinical trials and observational studies have suggested that disease severity, determined by maternal serum bile acid concentrations higher than the typical range, is associated with increased risk of adverse perinatal outcomes, including stillbirth, preterm birth, meconium staining of the amniotic fluid, fetal distress or asphyxia, and neonatal unit admission. We searched PubMed, Embase, and Web of Science (without language restrictions) for systematic reviews and meta-analyses published using the search terms “cholestasis”, “pregnancy”, “systematic review”, and “meta-analysis”. We found that the effect of drug treatments on intrahepatic cholestasis of pregnancy and perinatal outcomes stratified by disease severity has been previously assessed by meta-analysis, but no study has combined existing published literature to assess the effect of increased bile acid concentrations in women with intrahepatic cholestasis of pregnancy compared with uncomplicated pregnancy. We collected individual patient data from 27 studies, including two unpublished cohorts, with the aim of accurately determining how perinatal outcomes are associated with bile acid concentrations.

**Added value of this study**

This study is the first to do individual patient data analysis of perinatal outcomes and bile acid concentrations for women with intrahepatic cholestasis of pregnancy to show a clear association between women with the most severe disease (bile acids ≥100 μmol/L) and increased stillbirth risk (in singleton pregnancies) compared with those with milder disease and the background population.

**Implications of all the available evidence**

Our study shows that clinical management of women with intrahepatic cholestasis of pregnancy with singleton pregnancies can be stratified according to the maximum serum bile acid concentration, with the majority of women having bile acids lower than 100 μmol/L and, therefore, unlikely to have a higher risk of stillbirth than the background population. The women in our combined cohort were variably managed according to study centre, and the high proportion of iatrogenic preterm birth for women irrespective of peak bile acid concentration might have contributed to the lower prevalence of stillbirth observed for women with bile acids of less than 100 μmol/L than in some previous studies. Women with intrahepatic cholestasis of pregnancy should be managed according to their peak bile acid concentration, irrespective of treatment with ursodeoxycholic acid, provided repeated bile acid testing is done in women at low risk of stillbirth (ie, with bile acids <100 μmol/L). Our findings support the use of serum bile acid monitoring in cholestasis of pregnancy and provide strong support for ensuring that this test is widely available.

## Introduction

Intrahepatic cholestasis of pregnancy affects 0·1–2% of pregnant women;^1^, ^2^, ^3^, ^4^ it is diagnosed in women with gestational pruritus and increased serum bile acids, and can be complicated by preterm labour, fetal asphyxia, meconium-stained amniotic fluid, and stillbirth.^5^ Results from a large Swedish cohort showed that pregnancies in which the maternal serum bile acid concentration was of 40 μmol/L or more were more likely to be complicated by spontaneous preterm labour, meconium-stained amniotic fluid, and fetal asphyxia.^6^ A subsequent UK cohort study of pregnancy outcome in women with intrahepatic cholestasis of pregnancy with serum bile acids of 40 μmol/L or more supported these findings and also showed an association with intrauterine fetal death (adjusted odds ratio 3·05 [95% CI 1·65–5·63] when compared with data from 2205 women with uncomplicated singleton pregnancies in the UK.^7^ The association of high maternal serum bile acid concentrations with stillbirth is consistent with retrospective studies of women with intrahepatic cholestasis of pregnancy in the USA^8^ and Scandinavia.^9^ The 2007 stillbirth workshop^10^ included intrahepatic cholestasis of pregnancy as a medical disorder that can cause stillbirth in pregnancies when the maternal serum bile acid concentration is increased.^4^, ^6^, ^7^, ^11^

To our knowledge, no studies have been adequately powered to assess whether intrahepatic cholestasis of pregnancy -associated fetal death occurs above a certain bile acid threshold, and clinical guidelines are largely reliant upon expert consensus to determine the optimal management of affected women.^12^, ^13^ Clinicians often recommend management ranging from surveillance to iatrogenic delivery to prevent the subsequent risk of fetal death, at gestations typically between 36 weeks and 40 completed weeks, although the evidence behind this approach is scarce.^14^ Certainly, early delivery is associated with short-term neonatal problems and long-term issues with impairments in educational performance shown with even early-term birth.^15^, ^16^

We did a systematic review and meta-analysis to quantify the adverse perinatal effects of intrahepatic cholestasis of pregnancy in women with increased serum bile acid concentrations. We also aimed to determine whether elevated bile acid concentrations were associated with the risk of stillbirth. For the first aim, we extracted data from published studies reporting outcomes for women with intrahepatic cholestasis of pregnancy and control pregnancies; for the second aim, we did an individual patient data (IPD) meta-analysis to determine the relationships between biochemical markers and adverse perinatal outcomes.

## Methods

### Search strategy and selection criteria

In this systematic review and meta-analysis we searched Pubmed, Web of Science, and Embase databases using terms relating to intrahepatic cholestasis of pregnancy and perinatal outcomes, for articles published from database inception to June 1, 2018 (appendix). One additional study was identified by searching reference lists of selected studies and two unpublished cohorts from our research units were also included. Studies were selected for inclusion in one or both groups of the meta-analysis based on disease definition, including assessment of serum bile acid concentrations and reporting of perinatal outcomes. For the aggregate meta-analysis, studies including women with intrahepatic cholestasis of pregnancy and a control group were included, whereas for the IPD analysis, no control group was required. Otherwise, inclusion criteria were studies reporting bile acid concentrations and perinatal outcomes, which defined intrahepatic cholestasis of pregnancy based upon pruritus with raised serum bile acids with or without elevated aminotransferases. Case-control, cohort, and population-based studies, and randomised controlled trials were included. All studies were required to have ethical approval to share the data. Excluded studies included those at potential risk of increased bias, such as those with fewer than 30 participants, case reports, studies not comprising cohorts, or successive cases seen in a unit, and studies with high risk of bias from groups selected (eg, subgroup of babies with poor outcomes was explicitly excluded). Conference abstracts and Letters to the Editor without peer review were also excluded. We did not have language restrictions. When studies were not reported in English, electronic translation (Google Translate) was used to determine eligibility for inclusion in the meta-analysis, translating methods and results, with quality of translation determined by comprehensibility (CO); following abstract screening, only five manuscripts were translated. Study search and selection were done by two independent investigators (CO and AS), and a third (CW) arbitrated when any conflict occurred in the suitability of a study for inclusion. When studies recruited participants from the same hospital or population over the same time period, the study with the largest number of patients or reporting the most relevant outcome data was selected to avoid duplication.

Summary estimates were sought from studies with control data, to be analysed in an aggregate data meta-analysis. IPD were requested from corresponding and first or last authors by email and online platforms (Researchgate) in the language in which the manuscript was written. Authors who did not respond on two occasions were deemed to have not replied, and for all but one manuscript (contact details for other authors could not be found), at least two authors were contacted.

Respondents completed standardised spreadsheets, and data were standardised (units, equivalent decimal places, correct data entry, and categorisation of data allocation confirmed) and checked before statistical analysis (AS, CDI, and CO). Any disparities in the data received were discussed with the original authors.

Approval was obtained from the ethics committee of Hammersmith Hospitals National Health Service Trust (97/5197 and 08/H0707/21), London, UK, for inclusion of pseudoanonymised IPD. Approval was not required for the aggregate (extracted) data meta-analysis, because these data are anonymised and in the public domain; appropriate ethics approval was an inclusion criterion for all studies contained within the study.

### Unpublished data

Unpublished data from two UK hospitals (Imperial College Healthcare National Health Service [NHS] Trust and Guy's and St Thomas' NHS Foundation Trust, London, UK) were included in the IPD analysis. Women with intrahepatic cholestasis of pregnancy were identified through the antenatal clinics, maternity assessment unit, and antenatal wards, and recruited for an observational study, during which they prospectively completed health questionnaires and provided longitudinal blood samples. The study complied with the 1975 Declaration of Helsinki guidelines; participants provided written informed consent before inclusion. For women with more than one pregnancy during the recruitment period, only the first recorded pregnancy was included.

### Data analysis

Data from studies eligible for case-control aggregate data meta-analysis were extracted by two authors independently (CO and CDI), and any differences in reported values confirmed jointly. Maternal and perinatal outcomes were extracted, including maximum serum bile acid concentrations and liver function tests (maternal factors: parity, body-mass index (BMI), age, ethnicity, diabetes, hypertension, total bile acid concentration (peak), alanine aminotransferase concentration (peak), aspartate aminotransferase concentration (peak), bilirubin concentration (peak), ursodeoxycholic acid treatment, multifetal pregnancies, and caesarean section; perinatal outcomes: stillbirth, gestational age at delivery, preterm birth, iatrogenic preterm birth, spontaneous preterm birth, Apgar score <7 at 5 min, asphyxial events, meconium staining of the amniotic fluid, neonatal unit admission, and birthweight centile). When necessary, mean and SDs were estimated from medians using Wan's method.^17^ Data were compiled into a single dataset and any anomalies checked with the study authors.

Quality of the studies included in the aggregate data meta-analysis was determined using the Newcastle-Ottawa scale^18^ independently by two authors (CO and CDI). Any disparities in scoring were reviewed and consensus obtained following discussion. The quality of IPD studies was determined using the National Heart, Lung, and Blood Institute quality assessment tool for case series studies.^19^

We calculated mean differences and odds ratios [ORs] with GraphPad Prism (version 7.03).

We did a random effects meta-analysis with the DerSimonian and Laird method, presenting results as odds ratios or weighted mean differences for categorical and continuous variables. Meta-regression for confounders was done with restricted maximum likelihood with the Knapp Hartung modification for pruritic controls versus asymptomatic controls, multifetal pregnancy, and by study quality. We collected funnel plots and did the Harbord test to detect publication bias for each perinatal outcome reported, using log odds ratios for categorical variables. Variability between studies was determined by calculation of *I*^2^ and τ^2^ estimates of heterogeneity.

We did logistic regression for the IPD to obtain area under receiver operating characteristic (ROC) curves (ie, AUC) for the association between adverse perinatal outcomes and maximum serum total bile acids, alanine aminotransferase, aspartate aminotransferase, and bilirubin, for single and multifetal pregnancies, both individually and in combination.

Moreover, we did logistic regression using predefined total bile acid cutoffs of 20 μmol/L, 40 μmol/L, 60 μmol/L, 80 μmol/L, 100 μmol/L, 120 μmol/L, 140 μmol/L and more than 150 μmol/L to determine thresholds above which stillbirth increased; we used step functions, defining dummy variables using 0 for all values below the threshold and 1 for all values equal to or above the threshold. We used stepwise regression to simplify the model and remove unneeded cutpoints using Stata's stepwise regression command, which depends on the use of step functions, rather than non-overlapping categories.^20^, ^21^ We calculated hazard ratios (HRs) using Cox proportional model from a survival analysis to determine the proportion of stillbirth and spontaneous preterm birth by gestational week between bile acid concentration categories, analysing fetuses at risk for each gestational week.^22^ Post-hoc analysis of stillbirth proportions by bile acid category compared with reported national stillbirth proportions was done with the binomial probability test.

We did the analyses with Stata version 13. This study is registered with PROSPERO, number CRD42017069134.^23^

### Role of the funding source

The funders had no role in study design, data collection, data analysis, data interpretation, or writing of the report. The corresponding author had full access to all the data in the study and final responsibility for the decision to submit for publication.

## Results

**Figure 1:**
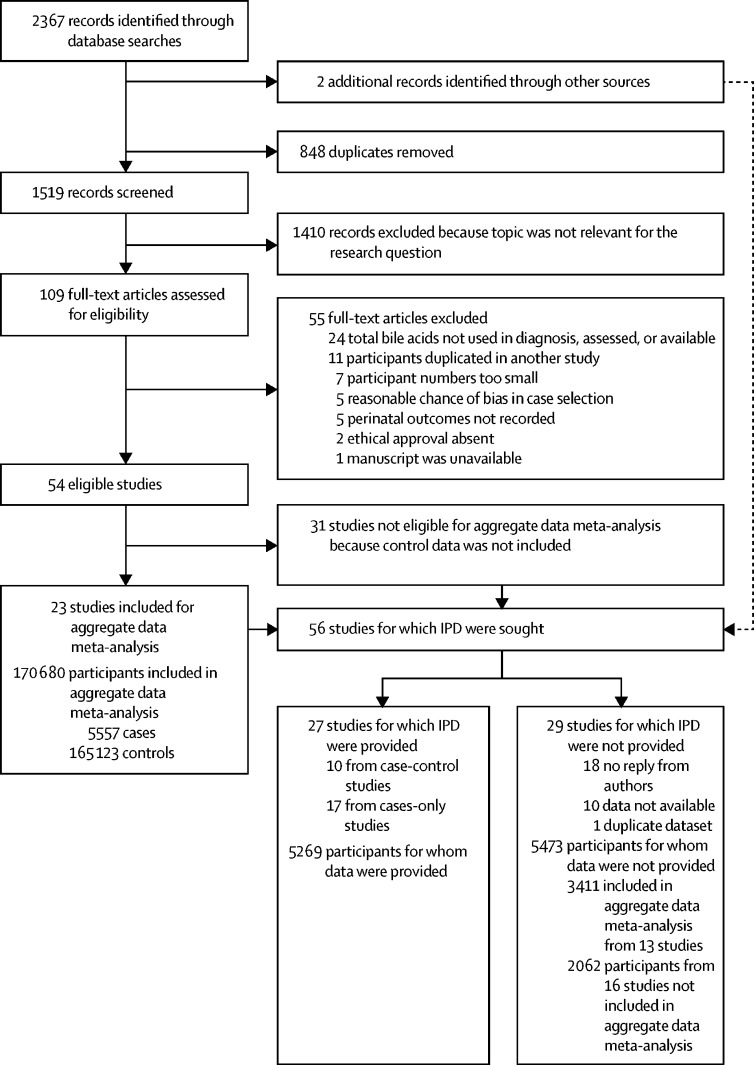
Flow chart of search results IPD=individual patient data.

23 studies reported intrahepatic cholestasis of pregnancy and control pregnant groups and fulfilled the inclusion criteria for the aggregate data meta-analysis comparing perinatal outcomes in intrahepatic cholestasis of pregnancy cases with controls (appendix). IPD was requested from these studies and 33 further studies (cases only without controls), including unpublished datasets from two hospital sites, with data received from 27 studies (ten case-control and 17 cases-only studies; figure 1; appendix).

Studies for which IPD were requested but were not received are listed in the appendix. Studies from 15 countries, from five continents, were included in the aggregate data meta-analysis, whereas IPD data were available from 14 countries, from five continents.

In the aggregate data meta-analysis, women with intrahepatic cholestasis of pregnancy had a slightly higher BMI than those with uncomplicated pregnancies (mean difference 1·6 kg/m^2^ [SD 0·2]) and were more likely to be of Asian ethnicity (appendix). Additionally, a higher proportion of women with intrahepatic cholestasis of pregnancy had pre-eclampsia and gestational diabetes than those without intrahepatic cholestasis of pregnancy.

Women in the IPD analysis had similar bile acid concentrations to intrahepatic cholestasis of pregnancy cases extracted from the systematic review, and the proportion treated with ursodeoxycholic acid was similar (appendix). Median fasting bile acid concentrations (n=1726, 23·0 μmol/L [IQR 14·7–41]) and prandial bile acid concentrations (n=2795, 32·0 μmol/L [19·0–61·5]) were similar with analysis restricted to those values from unselected cohorts of women with intrahepatic cholestasis of pregnancy.

**Figure 2:**
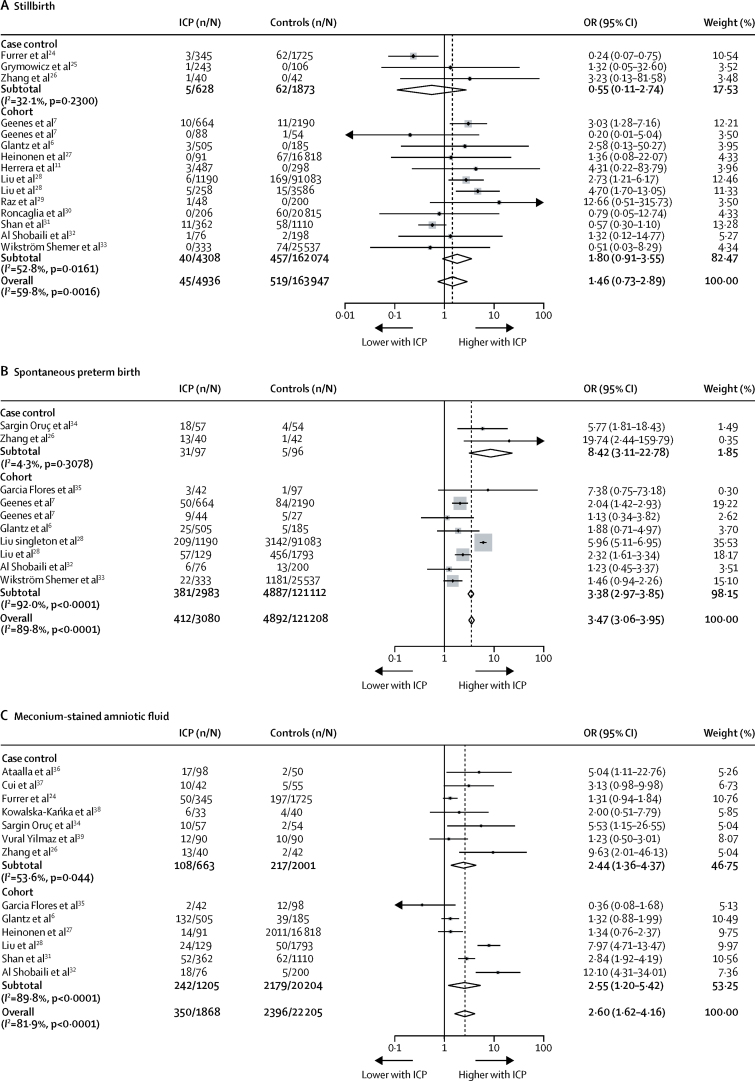
Forest plots of selected perinatal outcomes from aggregated patient data (A) Stillbirth; (B) spontaneous preterm birth; (C) meconium-stained amniotic fluid. Weights are from random effects analysis. ICP=intrahepatic cholestasis of pregnancy. OR=odds ratio.

Meta-analyses of data from the systematic review showed that, compared with controls, women with intrahepatic cholestasis of pregnancy had a higher risk of spontaneous preterm birth (OR 3·47 [95% CI 3·06–3·95; figure 2] and iatrogenic preterm birth (OR 3·65 [1·94 to 6·85]; appendix). Compared with controls, babies of intrahepatic cholestasis of pregnancy pregnancies were more likely to have meconium-stained amniotic fluid (OR 2·60 [95% CI 1·62–4·16]; figure 2) and be admitted to the neonatal unit (OR 2·12 [1·48–3·03]; appendix), but no difference was measured in birthweight centile (weighted mean difference 0·60 [95% CI −6·21 to 7·41]; appendix).

Findings from the meta-regression showed that study quality significantly contributed to the heterogeneity in results for meconium-stained amniotic fluid (p=0·0108); but multifetal pregnancies, controls comprising women with pruritus versus asymptomatic pregnancies, and study quality (according to Newcastle-Ottawa scale) did not significantly affect the results of other comparisons presented (appendix). The aggregate data meta-analysis showed the OR for stillbirth in women with intrahepatic cholestasis of pregnancy compared with controls to be 1·46 (95% CI 0·73–2·89; figure 2); with significant between study heterogeneity (τ^2^=0·81, p=0·0016; *I*^2^=59·8%). Although other perinatal outcomes did not reveal publication bias, this bias was significant for the meta-analysis of neonatal unit admission (p=0·037). Outlying studies were identified from the funnel plot, and removed for a subsequent analysis, which resulted in intrahepatic cholestasis of pregnancy having an attenuated effect on the increased risk of neonatal unit admission (OR 1·47 [1·03–2·10]; appendix).

**Figure 3:**
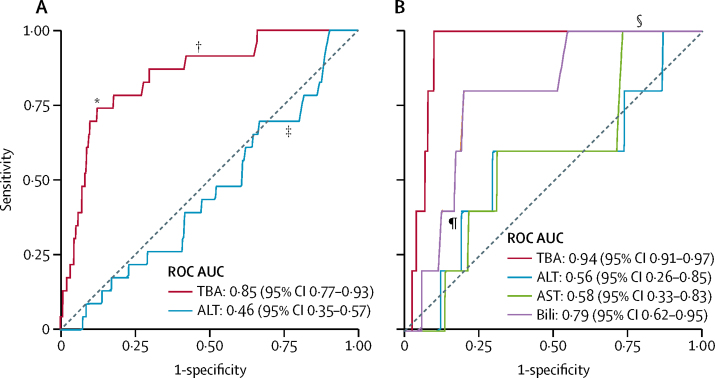
ROC curves for the association between stillbirth and serum biochemical markers for singleton pregnancies (A) Association between stillbirth and peak TBA and ALT concentrations for singleton pregnancies in a subset of women (n=3601) who had both biochemical tests. (B) Association between stillbirth and peak TBA, ALT, AST, and bilirubin concentrations for singleton pregnancies in a subset of women (n=1738) who had all four biochemical tests. ALT=alanine aminotransferase. AST=aspartate aminotransferase. AUC=area under the curve. ROC=receiver operating characteristic. TBA=total bile acid. *TBA=100 μmol/L. †TBA=40 μmol/L. ‡ALT=40 IU/L. §AST=40 IU/L. ¶Bilirubin=20 μmol/L.

**Figure 4:**
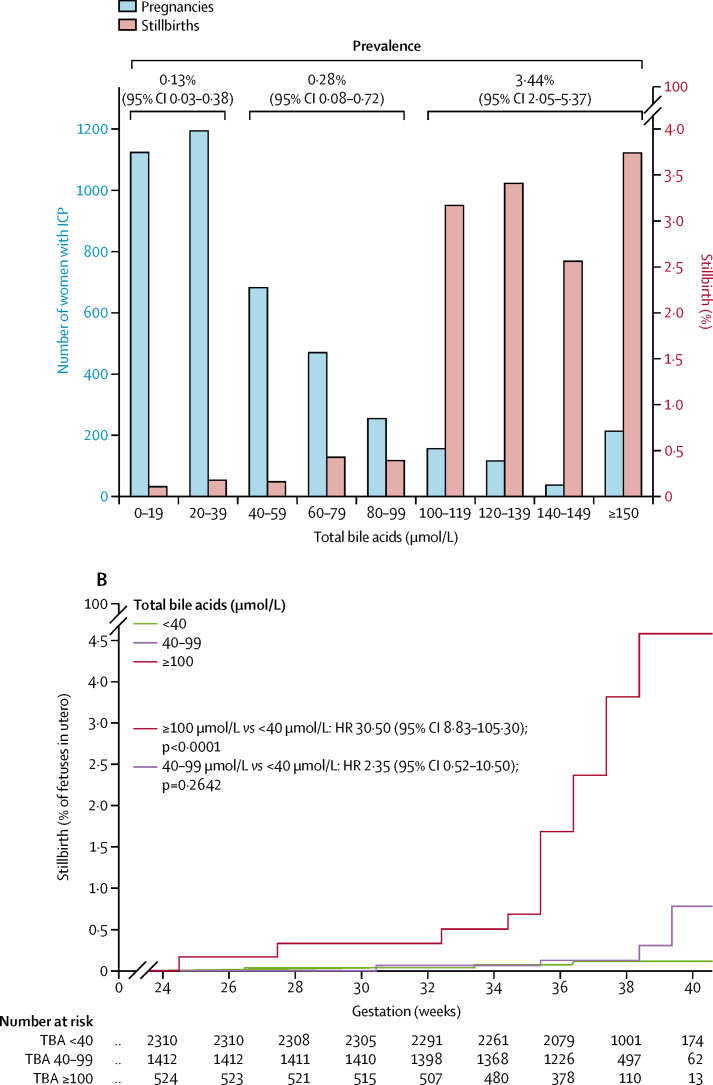
Proportion of stillbirths, number of pregnancies, and time-to-event analysis, by total bile acid concentrations in singleton pregnancies with intrahepatic cholestasis of pregnancy (A) Number of women with intrahepatic cholestasis of pregnancy (blue bars) and proportion of those women who had a stillbirth (red bars) by peak total bile acid category for women with singleton pregnancies. Stillbirth prevalence by total bile acid groups (<40 μmol/L, 40–99 μmol/L, and ≥100 μmol/L) is shown at the top of the graph. (B) Kaplan-Meir plot showing the proportion of fetuses in utero who were stillborn from 24 to 40 gestational weeks for singleton pregnancies. Data were analysed by completed gestational week categories, with alterations plotted mid-week to reflect uncertainty by individual day of change. Data are not shown from 40 weeks because of the low remaining numbers of fetuses in utero. HR=hazard ratio. ICP=intrahepatic cholestasis of pregnancy.

IPD analysis showed that total bile acid concentrations were more highly predictive of stillbirth for singleton pregnancies than the other biomarkers assessed (ROC AUC 0·85 [95% CI 0·77–0·93]; figure 3A), whereas the associations between stillbirth and alanine aminotransferase (ROC AUC 0·46 [95% CI 0·35–0·57]; figure 3A) and aspartate aminotransferase (ROC AUC 0·58 [0·33–0·83]; figure 3B) were lower than for total bile acid; bilirubin was also less predictive of stillbirth than was total bile acid (ROC AUC 0·79 [0·62 to 0·95]; figure 3B, table). These associations were not present in multifetal pregnancies, although the smaller numbers of multifetal pregnancies than singleton pregnancies reduced the reliability of these results (appendix). No other adverse perinatal outcomes were highly associated with any biochemical marker assessed (table). A sensitivity analysis excluding unpublished studies made no difference to these conclusions (appendix). Treatment with ursodeoxycholic acid did not significantly affect this association (appendix). To assess whether a threshold of total bile acid concentration associated with an increased risk of stillbirth could be defined, we did stepwise logistic regression between bile acid categories at 20 μmol/L intervals; for women with singleton pregnancies, total bile acids of 100 μmol/L or more were significantly associated with an increased risk of stillbirth (p<0·0001). The majority of women with intrahepatic cholestasis of pregnancy had maximum total bile acids of less than 100 μmol/L (figure 4A; appendix); the increased risk of stillbirth thus was associated with a minority of women with intrahepatic cholestasis of pregnancy. The prevalence of stillbirth in singleton pregnancies was lowest for women with serum total bile acids of less than 40 μmol/L after 24 gestational weeks, and highest for those with total bile acids of 100 μmol/L or higher (figure 4A). A time-to-event analysis by each gestational week the fetus remained in utero for these bile acid categories (fetus at risk) for singleton pregnancies showed that the HR for stillbirth in women with bile acids of 40–99 μmol/L was not significant when compared with women with total bile acids of less than 40 μmol/L, whereas the HR for women with bile acids of 100 μmol/L or more was significant (figure 4B; appendix). The risk of stillbirth increased as gestation progressed (figure 4B). Similar results were obtained with a sensitivity analysis, assuming that all iatrogenic deliveries in the original survival analysis would not have been followed by a stillbirth before 40 gestational weeks (appendix).

**Table:** Summary of individual patient data associations between serum biochemistry and adverse perinatal outcome for singleton pregnancies

	**Bile acids**		**Alanine aminotransferase**		**Aspartate aminotransferase**		**Bilirubin**	
	n/N (%)	ROC AUC (95% CI)	n/N (%)	ROC AUC (95% CI)	n/N (%)	ROC AUC (95% CI)	n/N (%)	ROC AUC (95% CI)
Stillbirth	25/4269 (1%)	0·83 (0·74–0·92)	22/3668 (1%)	0·46 (0·35–0·57)	17/3071 (1%)	0·49 (0·36–0·62)	13/2425 (1%)	0·57 (0·42–0·72)
Preterm birth	1256/4378 (29%)	0·60 (0·58–0·63)	583/1836 (32%)	0·55 (0·52–0·57)	583/1836 (32%)	0·54 (0·51–0·57)	583/1836 (32%)	0·57 (0·54–0·60)
Spontaneous preterm birth	383/4316 (9%)	0·61 (0·58–0·64)	141/1791 (8%)	0·59 (0·54–0·64)	141/1791 (8%)	0·59 (0·54–0·65)	141/1791 (8%)	0·57 (0·51–0·62)
Iatrogenic preterm birth	817/4316 (19%)	0·58 (0·55–0·60)	397/1791 (22%)	0·53 (0·50–0·56)	397/1791 (22%)	0·52 (0·50–0·55)	397/1791 (22%)	0·56 (0·53–0·59)
Meconium stained amniotic fluid	588/4032 (15%)	0·62 (0·59–0·64)	243/1605 (15%)	0·59 (0·55–0·63)	243/1605 (15%)	0·59 (0·56–0·63)	243/1605 (15%)	0·57 (0·53–0·61)
Non-reassuring heart rate monitoring	588/3057 (19%)	0·58 (0·55–0·60)	192/1379 (14%)	0·50 (0·46–0·55)	192/1379 (14%)	0·53 (0·49–0·58)	192/1379 (14%)	0·54 (0·49–0·58)
Apgar score <7 at 5 min	90/4181 (2%)	0·65 (0·58–0·71)	32/1698 (2%)	0·45 (0·36–0·54)	32/1698 (2%)	0·49 (0·40–0·58)	32/1698 (2%)	0·51 (0·40–0·62)
Umbilical cord arterial blood pH <7·0	1/2029 (1%)	0·68 (0·53–0·82)	6/630 (1%)	0·48 (0·21–0·76)	6/630 (1%)	0·49 (0·22–0·75)	6/630 (1%)	0·52 (0·23–0·81)
Neonatal unit admission	798/4014 (20%)	0·55 (0·52–0·57)	182/1533 (12%)	0·57 (0·53–0·62)	182/1533 (12%)	0·58 (0·54–0·63)	182/1533 (12%)	0·55 (0·51–0·60)
Neonatal death	7/2888 (<1%)	0·62 (0·38–0·86)	5/1391 (<1%)	0·56 (0·31–0·84)	5/1391 (<1%)	0·62 (0·38–0·87)	5/1391 (<1%)	0·68 (0·53–0·84)

ROC AUC=receiver operating characteristic area under curve.

To establish whether risk of stillbirth in women with total bile acids of less than 100 μmol/L was increased compared with the background population risk, we used published data on the prevalence of national stillbirth as comparator groups (appendix) in a weighted analysis.^40^ We found no increased stillbirth risk for women with singleton intrahepatic cholestasis of pregnancy who were included in our IPD analysis with total bile acids of less than 40 μmol/L or 40–99 μmol/L when compared with the pooled national prevalence of stillbirth from 2000 (0·42%) or 2015 (0·33%).

**Figure 5:**
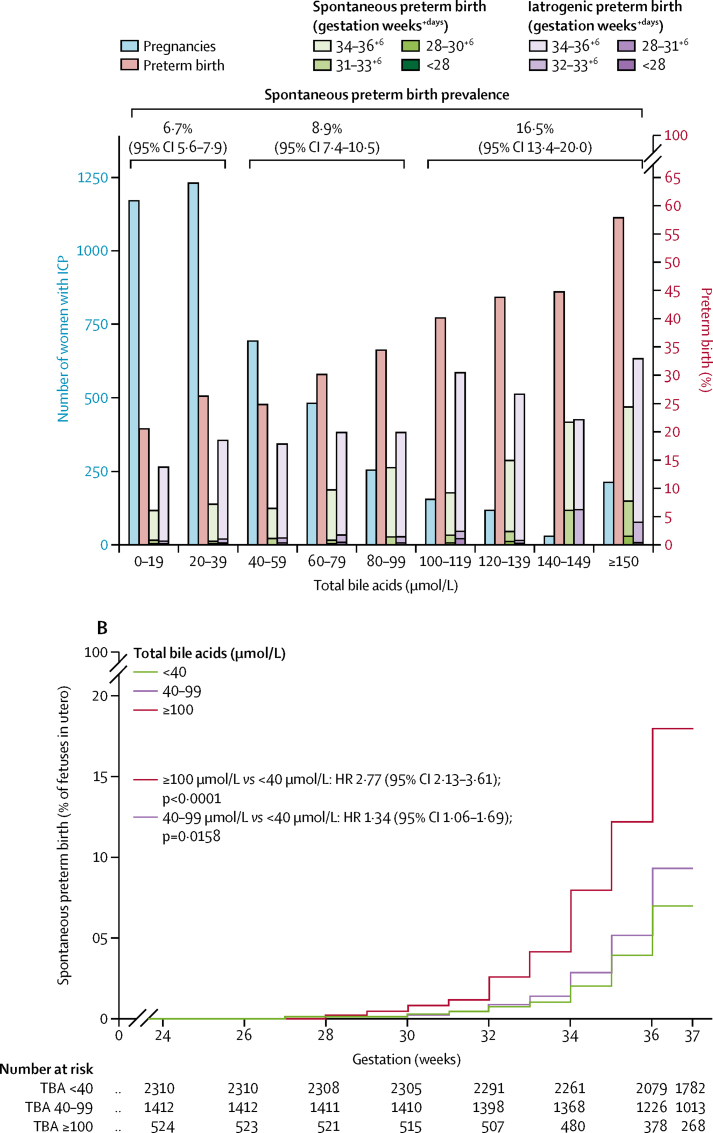
Proportion of preterm births, number of pregnancies, and time-to-event analysis, by total bile acid concentrations in singleton pregnancies with intrahepatic cholestasis of pregnancy (A) Number of women with intrahepatic cholestasis of pregnancy (blue bars), and proportion of those women with overall preterm birth (red bars), spontaneous preterm birth by gestational week (green bars), and iatrogenic preterm birth by gestational week (purple bars), by peak total bile acid category for women with singleton pregnancies. Spontaneous preterm birth (more clinically relevant than overall preterm birth because it is not clinician dependent) prevalence by total bile acid groups (<40 μmol/L, 40–99 μmol/L, and ≥100 μmol/L or more) is shown at the top of the graph. (B) Kaplan-Meir plot showing the proportion of fetuses in utero who underwent spontaneous preterm birth from 24 to 37 gestational weeks for singleton pregnancies (birth from 37 gestational weeks is not considered preterm). Data were analysed by completed gestational week categories, with alterations plotted mid-week to reflect uncertainty by individual day of change. HR=hazard ratio. ICP=intrahepatic cholestasis of pregnancy.

Results from the aggregate data meta-analysis showed that women with intrahepatic cholestasis of pregnancy had higher ORs of preterm birth (OR 3·54 [95% CI 2·72–4·62]; appendix), but significant heterogeneity was observed between studies, particularly for iatrogenic preterm birth. Although the risk of preterm birth in women with intrahepatic cholestasis of pregnancy was significant, no strong association was measured between preterm birth and serum biochemistry (table). However, an increase in preterm birth was evident with more marked elevations of total bile acid concentrations (figure 5A). Increasing HRs for spontaneous preterm birth by gestational week were seen with increasing bile acid category (figure 5B; appendix).

The prevalence of iatrogenic preterm birth was high for all categories of bile acid concentration (<40 μmol/L, 16·5% [95% CI 15·1–18·0]; 40–99 μmol/L, 19·1% [17·1–21·1]; and ≥100 μmol/L, 30·5% [26·8–34·6]). The majority of multifetal pregnancies were born preterm (appendix).

## Discussion

This meta-analysis provides evidence that intrahepatic cholestasis of pregnancy is associated with adverse perinatal outcomes, with a significantly increased risk of stillbirth for women with serum total bile acids of 100 μmol/L or more. Iatrogenic preterm birth is a major contributor to the higher prevalence of preterm birth in intrahepatic cholestasis of pregnancy (for women with any bile acid concentration) than in control pregnancies, although the prevalence of spontaneous preterm birth increases with higher total bile acid concentrations.

One of the strengths of this meta-analysis is the careful definition of cholestatic disease to include serum total bile acids in the diagnostic criteria, and supporting IPD providing the largest combined cohort to date of women with intrahepatic cholestasis of pregnancy to investigate the associations between biochemical abnormalities and adverse pregnancy outcomes. Given that stillbirth is a rare outcome, a cohort of this size is necessary to obtain reliable conclusions. A further strength is the collaborative use of data from multiple studies and centres. In particular, authors of smaller studies frequently state that larger studies are needed to substantiate findings with regard to stillbirth. Using previously collected data avoids research waste, as highlighted in the *Lancet* Series published in 2014.^41^ One limitation of the aggregate data meta-analysis is the inconsistency in the definition of perinatal outcomes of neonatal asphyxia, resulting in difficulty with comparison of studies. The CoRe Outcomes in Women's and Newborn health initiative^42^ intends to address these difficulties, and with more widespread adoption, will enable meta-analyses to appraise such study outcomes.

A further challenge of this study was the inability to adjust results by all confounders because of incomplete reporting. Although we were able to do meta-regression for study-level confounders (multifetal pregnancy proportions and study quality), individual patient characteristics, such as coexistent pre-eclampsia and gestational diabetes, were not accounted for. Proportions of both diseases were higher in the populations of women with intrahepatic cholestasis of pregnancy compared with controls, as expected from previous literature.^43^, ^44^ Previous studies have reported an association of maternal comorbidities (such as pre-eclampsia and gestational diabetes) with stillbirth in women with intrahepatic cholestasis of pregnancy and bile acids of more than 40 μmol/L.^7^ Since increased bile acids are not a feature of either of these pregnancy complications, and the association between intrahepatic cholestasis of pregnancy and stillbirth has been reported with and without the comorbidities, this association is likely to be true, although the possibility of additive factors contributing to stillbirth risk remains likely.

Other small studies have previously suggested an association between intrahepatic cholestasis of pregnancy and adverse perinatal risks, but their clinical implications have been limited by their size.^11^, ^27^, ^31^, ^45^ Cui and colleagues^46^ did a meta-analysis with extracted data from women with intrahepatic cholestasis of pregnancy and showed that adverse perinatal outcomes (eg, preterm birth) were increased in women with bile acids greater than 40 μmol/L compared with women with lower bile acids, but did not report effects on stillbirth risks. Thus, the clear bile acid threshold of 100 μmol/L beneath which the prevalence of stillbirth was not increased is a novel and important finding in our study. This threshold was reached using bile acid concentrations obtained with differing methods (appendix), yet interlaboratory quality control procedures were likely to minimise the unreliability of measurements, and our findings reflect everyday clinical results.

Results from two studies have suggested the optimal delivery time for women with intrahepatic cholestasis of pregnancy to be 36 gestational weeks.^47^, ^48^ Puljic and colleagues^47^ calculated a composite mortality risk by gestational week with data registries for 5545 Californian women with intrahepatic cholestasis of pregnancy and matched controls, identifying 36 weeks as the optimal delivery week to prevent stillbirth or neonatal death for singleton pregnancies. Their study reported an overall stillbirth prevalence of 0·64% for women with intrahepatic cholestasis of pregnancy and singleton pregnancies (similar to the 0·59% [95% CI 0·39–0·87] that we report in the IPD analysis), although the authors did not stratify women according to disease severity and did not account for additional neonatal morbidity secondary to prematurity. Lo and colleagues^48^ also identified 36 weeks to be the optimal gestation for delivery using computer modelling to determine subsequent maternal and child quality-adjusted life-years achieved, compared with delivery at other gestations between 35 and 38 weeks. They estimated the prevalence of stillbirth in women with intrahepatic cholestasis of pregnancy to be 1·74%, and their model was robust to a stillbirth prevalence that was 30% higher (ie, up to 2·26%) and 60% lower (ie, down to 0·70%) than this estimate. However, when stratifying women according to their maximum bile acid concentration of greater or less than 100 μmol/L in our IPD analysis, stillbirth prevalence was outside of these ranges (0·13–0·28% for <100 μmol/L and 3·44% for ≥100 μmol/L), reducing the applicability of their model to our population.

Our study identifies a clear association between bile acid concentrations and stillbirth for singleton pregnancies. Importantly, the stillbirth risk was increased in women with total bile acid concentrations of 100 μmol/L or more at any point in the pregnancy. The HR for stillbirth increased with gestation time; the prevalence of stillbirth for all bile acid groups is less than 1% before 35 completed weeks of pregnancy. For the women in our dataset with peak bile acid concentrations of less than 100 μmol/L and singleton pregnancies, we found no increase in stillbirth compared with the background population risk before 39 weeks' gestation; however, the 25·3% preterm birth prevalence in this group, the largest proportion of which was iatrogenic, might have contributed to prevention of later stillbirth. The so-called fetus at risk approach used to determine HRs of stillbirth between women of different bile acid categories accounts for this background at risk approach, in part. This approach is more reliable when the denominator (number of fetuses in utero at the start of that gestational week) is large than when it is small (eg, in our study, at 24 weeks, over 500 women were included with peak bile acids in the pregnancy of >100 μmol/L; however, at 39 weeks, only 13 remained in the study). Since the majority of babies were born by 39 weeks' gestation, we did not present stillbirth beyond that gestation because the number at risk would be too low for the results to be clinically meaningful and the uncertainty rendered the findings clinically unreliable. However, we recognise the limitations of our approach; such biases are inevitable in a non-randomised study and bias correction is not always possible. Although the most reliable solution is to do a randomised controlled trial addressing the question, a pilot timing-of-delivery trial in women with intrahepatic cholestasis of pregnancy concluded that a randomised trial was unlikely to be feasible^49^ and the challenges of designing such a trial have been widely recognised because of the rarity of stillbirth as a pregnancy outcome.^50^ A systematic review by Henderson and colleagues^14^ concluded that evidence was insufficient to support the practice of active management (ie, proactively arranging delivery) for intrahepatic cholestasis of pregnancy. The increased risk of stillbirth in women with bile acids of >100 μmol/L or more suggests that active management (most likely to be undertaken in women with the highest concentrations of bile acids) does not completely eliminate the risk of stillbirth. Although our data cannot confirm that the risk of stillbirth is not increased for women with bile acids of less than 100 μmol/L compared with the background population if not actively managed, these women were unlikely to be managed more proactively than those with bile acids of 100 μmol/L or more. Women included in our IPD cohort were unlikely to have not received antenatal care, which in itself is associated with an increased risk for antepartum stillbirth,^51^ and this confounder might have contributed to the low prevalence of stillbirth observed for women with bile acids of less than 100 μmol/L in comparison with national stillbirth proportions.

Peak total bile acid concentrations were associated with stillbirth risk, whether or not women were taking ursodeoxycholic acid. This analysis was not designed to determine whether ursodeoxycholic acid treatment can reduce the risk of stillbirth, which would be best addressed by randomised controlled trials such as the PITCHES randomised controlled trial, which is in progress in the UK.^52^

Two suggested models of fetal demise in intrahepatic cholestasis of pregnancy are consistent with evidence indicating that high bile acids contribute to the causes of adverse outcomes: increased bile acids are associated with fetal cardiac arrhythmia and placental vessel spasm.^53^, ^54^ Without data on the timings of peak bile acid concentration and associated stillbirth gestation, this study cannot provide further mechanistic evidence to support these models; as such, we suggest managing women with intrahepatic cholestasis of pregnancy and singleton pregnancies on the basis of their peak bile acid concentration. Because bile acids can change rapidly with advancing gestation,^55^ regular monitoring of serum total bile acids (eg, weekly) is needed to reassess risk. Bile acids might increase postprandially; the conclusions of the IPD meta-analysis were based on a mixed population of sampling protocols, with the majority of studies using non-fasting measurements, but the median bile acid concentrations were similar whether measured when fasting or postprandially.

In summary, this study has clarified the adverse pregnancy outcomes associated with intrahepatic cholestasis of pregnancy and has identified that women with serum bile acids of 100 μmol/L or more have a significantly increased risk of stillbirth. Future research should target mechanistic explanations for the increased risk of stillbirth in intrahepatic cholestasis of pregnancy and the potential of specific treatments to prevent fetal death.
