# Department of Error

**DOI:** 10.1016/S0140-6736(19)30504-5

**Published:** 2019-03-16

*Ovadia C, Seed PT, Sklavounos A, et al. Association of adverse perinatal outcomes of intrahepatic cholestasis of pregnancy with biochemical markers: results of aggregate and individual patient data meta-analyses.* Lancet *2019; **393:** 899–909*—In this Article, the correct author name is Chiara Di Ilio; Prof Laura Bull highest degree is PhD, not MD; and, in the Summary, under Results, stillbirth occurred in 0·91% of pregnancies, not in 0·83% of pregnancies. These corrections have been made as of March 14, 2019.

