# Supplementary material for: Association of adverse perinatal outcomes of intrahepatic cholestasis of pregnancy with biochemical markers: results of aggregate and individual patient data meta-analyses
Source: Lancet. 2019 Mar 2;393(10174):899–909. doi: 10.1016/S0140-6736(18)31877-4 (PMC6396441; doi:10.1016/S0140-6736(18)31877-4)
Supplement: Supplementary appendix [file mmc1.pdf]

THE LANCET

Supplementary appendix

This appendix formed part of the original submission and has been peer reviewed. We post it as supplied by the authors.

Supplement to: Ovadia C, Seed PT, Sklavounos A, et al. Association of adverse perinatal outcomes of intrahepatic cholestasis of pregnancy with biochemical markers: results of aggregate and individual patient data meta-analyses. *Lancet* 2019; published online Feb 14. http://dx.doi.org/10.1016/S0140-6736(18)31877-4.

Appendix contents

Table 1. Selection criteria used for identification of relevant literature 2
Table 2. Description of studies used for aggregate data meta-analysis 3
Table 3. Studies used for individual patient data analysis 7
Table 4. Studies not included in individual patient data analysis for which data were Requested 12
Table 5. Demographic and pregnancy characteristics of women included in the systematic review and IPD analysis 13
Table 6. Description of ICP disease 14
Table 7. Summary table of results – aggregate data case-control comparisons 15
Table 8. Summary of IPD associations between serum biochemistry and adverse perinatal outcomes for multifetal pregnancies 16
Table 9. Sensitivity analysis of IPD associations between serum biochemistry and adverse perinatal outcomes for singleton pregnancies excluding unpublished studies 17
Table 10. National rates of stillbirth from 28 gestational weeks for studies included in the individual patient data analysis 18
Figure 1. Forest plots of selected perinatal outcomes from aggregated patient data 19
Figure 2. Correction for publication bias in meta-analysis of neonatal unit admission rates in intrahepatic cholestasis of pregnancy 20
Figure 3. Receiver operating curves for association between stillbirth and serum biochemical markers for multifetal pregnancies 21
Figure 4. Receiver operating characteristic curve for the association between total bile acid concentrations and stillbirth for women treated and untreated with ursodeoxycholic acid 22
Figure 5. Proportion and incidence of stillbirths and total number of pregnancies by total bile acid concentrations in multifetal pregnancies with intrahepatic cholestasis of pregnancy 23
Figure 6. Stillbirth rate by number of fetuses *in utero* for singleton pregnancies 24
Figure 7. Sensitivity analysis of stillbirth rate by number of fetuses *in utero* for singleton pregnancies 25
Figure 8. Spontaneous preterm birth rate by number of fetuses *in utero* for singleton pregnancies 26
Figure 9. Proportion and incidence of preterm births and total number of pregnancies by total bile acid concentrations in multifetal pregnancies with intrahepatic cholestasis of pregnancy 27
References 28

Table 1. Selection criteria used for identification of relevant literature

ICP-related search terms	"cholestasis, intrahepatic, of pregnancy", "pregnancy-related cholestasis", "pregnancy related cholestasis", "recurrent intrahepatic cholestasis of pregnancy", "obstetric cholestasis", "cholestasis, intrahepatic of pregnancy", "cholestasis, pregnancy-related", "familial intrahepatic cholestasis of pregnancy", "intrahepatic cholestasis of pregnancy"
Perinatal outcome-related search terms	"pregnancy outcome", "fetal outcome", "fetal distress", "obstetric labor complication*", "pregnancy, high*risk", "delivery, obstetric", "labor, obstetric", "live birth", "obstetric labor, premature", "premature birth", "caesarean section", "abortion, spontaneous", "stillbirth", "fetal death", "infant mortality", "maternal mortality", "perinatal mortality", "gestational age", "infant, low birth weight", "apgar score", "pregnancy outcome*", "pregnancy complication", "obstetric outcome*", "obstetric complication*", "normal birth*", "live birth*", "premature birth*", "preterm birth*", "preterm deliver*", "born preterm", "cesarean*", "c-section", "caesarean*", "miscarriage*", "stillbirth*", "intrauterine death*", "neonatal death*", "postpartum haemorrhage*", "postpartum hemorrhage*", "postpartum complication*", "special care baby unit admission*", "SCBU admission*", "neonatal intensive care unit admission*", "NICU admission*", "neonatal unit*", "NNU admission*", "small for gestational age", "SGA", "intra-uterine growth restriction", "IUGR", "Apgar"
Inclusion criteria	Prospective case-control studies, prospective cohort studies, retrospective cohort studies, population based studies, and randomised controlled trials that reported bile acid concentrations and perinatal outcomes (at least one of stillbirth, preterm birth, neonatal unit admission, and meconium stained amniotic fluid)<br>Personal communications of unpublished cohorts were included in the IPD meta-analysis<br>ICP diagnosis based upon pruritus and elevated serum bile acid concentrations with or without raised liver transaminase concentrations<br>Ethical approval to share the data
Exclusion criteria	Case reports, studies not comprising cohorts, or successive cases seen in a unit, studies with high risk of bias from groups selected (e.g. where a sub-group of babies with poor outcomes were explicitly excluded)<br>Studies with 30 or fewer study participants<br>Diagnosis of ICP not using serum bile acid concentrations<br>Abstracts, Letters to the Editor without peer review<br>Studies that did not report serum bile acid concentrations or any perinatal outcomes

ICP: intrahepatic cholestasis of pregnancy; IPD: individual patient data. Prospective studies were recruited throughout the study period, with results accumulated in real time, whilst retrospective studies were defined as those recruited following conclusion of the associated pregnancies. Case-control studies were those recruiting affected women with ICP and matched control (unaffected) women, whilst cohort studies recruited a population of women (national / regional / local) and observed their outcomes. Randomised controlled trials compared the use of an intervention between groups of affected women.

Table 2. Description of studies used for aggregate data meta-analysis

	Nº of cases	Nº of controls	Country	Design	Multifetal pregnancies	Definition of ICP used	Data quality (NOS)	Data collection period	Major predictors reported	Outcome measures reported
Al Shobaili et al (2011)[1]	76	200	Saudi Arabia	Prospective cohort	2·6% ICP 2·0% controls	Pruritus onset in pregnancy, ↑TBA, no pre-gestational skin disease, no other cause of pruritus, gall bladder, or liver disease	4	2008-2010	TBA, ALT, bilirubin, US doppler indices	Apgar score, GA delivery, IUFD, MOD, MSAF, NNU, NRHRM, PPH, PTB, RDS, UC arterial pH
Ataalla et al (2016)[2]	98	50	Egypt	Prospective case-control	n/a	Pruritus without a rash (except excoriative), ↑TBA, no other liver disease, skin disease, allergic disorder, cholelithiasis, or hepatic viral infection	4	2011-2013	Pruritus severity, TBA, ALT, AST, bilirubin, ALP, alb	Apgar score, fetal distress, fetal echocardiogram, GA delivery, MSAF, neonatal bilirubin, neonatal TBA, RDS, sepsis
Castaño et al (2006)[3]	41	30	Argentina	Prospective case-control	n/a	Pruritus, ↑TBA, and ↑ALT/AST after 20/40, no other autoimmune disease, moderate-severe alcohol intake, HIV infection, Hepatitis A, B, or C infection, skin disease, or biliary obstruction, post-partum normalisation	4	2004-2005	Pruritus severity, TBA, individual BA, ALT, AST, bilirubin, GGT, ALP	Apgar score, BW, GA delivery, SGA
Cui et al (2018)[4]	42	55	China	Prospective case-control	21·4% ICP 3·6% controls	As per RCOG Guideline 2nd edition: i.e. gestational pruritus, ↑TBA and/or ↑ALT/AST, postpartum resolution, no other cause of itching or liver dysfunction[5]	5	2012-2015	TBA, individual BA, ALT, AST, bilirubin, GGT, ALP	BW, CS, EBL, GA delivery, MSAF
Furrer et al (2016)[6]	345	1725	Switzerland	Retrospective case-control	22·9% ICP 22·8% controls	Database coding, no other liver dysfunction	8	2004-2014	TBA	Apgar score, blood transfusion, BW, EBL, GA delivery, Hb pre- and post- delivery, IOL, MOD, MSAF, NNU, perinatal death, UC arterial pH

Garcia-Flores et al (2015)[7]	47	98	Spain	Prospective cohort	23·4% ICP<br>4·1% controls	Pruritus, ↑TBA, no other cause for raised bile acids	8	2012-2014	TBA, ALT, AST	Apgar score, birth biometry (e.g. BW, BW centile), GA delivery, IOL, MOD, MSAF, neonatal morbidity, NNU, NRFHR, SPTB, UC arterial pH
Geenes et al (2014)[8]	713	2232	UK	Prospective population cohort	6·2% ICP<br>1·2% controls	Pruritus without a rash, severe ICP: TBA≥ 40μmol/L	8	2010-2011	TBA, ALT, AST, bilirubin, GGT	Apgar score, BW, BW centile, GA delivery, IPTB, IUFD, MOD, MSAF, NNU, SPTB
Glantz et al (2004)[9]	505	185	Sweden	Prospective cohort	5·3% ICP<br>n/a controls	Gestational pruritus without dermatological cause, ↑TBA	8	1999-2002	Pruritus severity, TBA, ALT, AST, bilirubin	Asphyxial events, EBL, GA delivery, IOL, IUFD, MOD, MS placenta or membranes, MSAF, PTB
Grymowicz et al (2016)[10]	157	46	Poland	Prospective case-control	19·7% ICP<br>3·0% controls	Pruritus onset after 20/40, ↑TBA or ALT/AST, no other cause of pruritus, liver disease, or dermatological disease	8	2005-2006	Pruritus severity, TBA, ALT, AST, bilirubin	Apgar score, asphyxial events, BW, congenital anomalies, GA delivery, ICH, IOL, MOD, MSAF ↑ neonatal bilirubin, NNU, RDS
Heinonen and Kirkinen (1999)[11]	91	16818	Finland	Prospective cohort	0%	Pruritus, ↑TBA, and ALT/AST, post-partum normalisation, no other dermatological condition, or viral hepatitis, normal liver and biliary tract ultrasound	7	1990-1996	LFTs	Anaemia, Apgar score, APH, IUFD, LBW, MOD, MSAF, NNU, NRHRM, PET, placenta praevia, placental abruption, placental/fetal mass, PTB, Rh immunisation, SGA, UC venous pH
Herrera et al (2018)[12]	487	298	USA	Retrospective cohort	7·8% ICP<br>5·4% controls	ICD9 codes, ↑TBA	8	2005-2015	TBA, ALT, AST	Apgar score, CS, GA delivery, MSAF, neonatal bilirubin, neonatal morbidity, NNU, perinatal death, RDS, SGA, SPTB, UC arterial pH
Kebapcilar et al (2010)[13]	40	40	Turkey	Prospective case-control	n/a	Pruritus onset after 20/40, ↑TBA, no other liver disease, or skin disease	5	2008-2009	TBA, ALT, AST, ALP, lipid, and coagulation profiles	Apgar score, GA delivery, IUFD, US doppler indices
Kowalska-Kańka et al (2013)[14]	33	40	Poland	Case-control	24·2% ICP<br>15·0% controls	Pruritus, ↑TBA, and ↑ALT/AST, no other cause of pruritus, liver or biliary tract disorder	5	2009-2011	Pruritus severity, TBA, ALT, AST, bilirubin, EPO, FBC, coagulation profile	Apgar score, BW, GA delivery, NRHRM, UC arterial pH, pCO_2, pO_2, and BE, US indices
Liu et al (2016)[15]	1448	94 669	China	Retrospective population cohort	9·8% ICP<br>1·9% controls	Pruritus, ↑TBA	8	2006-2014	TBA	Apgar score, BW, GA delivery, GDM, IOL, IUFD, LGA, MSAF, NND, NNU, PET, PTB, RDS, SGA

Oztas et al (2014)[16]	117	100	Turkey	Prospective case-control	0%	Pruritus, ↑TBA, and/or ALT/AST, no other dermatological disease, liver disease, renal disease, cholelithiasis, or viral infection affecting the liver	6	2012-2014	TBA, ALT, AST, bilirubin, GGT, FBC, coagulation profile	Apgar score, BW, GA delivery, IUFD, IUGR, NNU, oligohydramnios, PET, PTB
Raz et al (2015)[17]	78	300	Israel	Retrospective cohort	30·8% ICP 33·3% controls	Pruritus, ↑TBA, and LFTs, no other viral infection affecting the liver, liver disease, or biliary disease	6	2008 -2014	TBA, LFTs, FBC, proteinuria, PCR, BP	Apgar score, BW, BW centile, GA delivery, IOL, IUFD, MOD, MSAF, neonatal complications, NRHRM, PET, PTB, UC blood pH
Roncaglia et al (2002)[18]	206	20815	Italy	Prospective cohort	5·8% ICP n/a controls	Pruritus onset in 2nd/3rd trimesters, post-partum normalisation, ↑TBA or ↑ALT/AST, no other cause of pruritus	4	1989-1997	Pruritus severity TBA, ALT, AST, bilirubin	Apgar score, GA delivery, IOL, IUFD, meconium aspiration, MOD, MSAF, oligohydramnios, PTB SGA, UC arterial pH
Sargin Oruç et al (2014)[19]	57	54	Turkey	Prospective case-control	0%	Pruritus, ↑TBA, no other liver disease, skin, or allergic disorder, symptomatic cholelithiasis, or ongoing viral infection affecting the liver	7	2012	TBA, ALT, AST	Apgar score, BW, fetal asphyxia, GA delivery, IOL, IUFD, MSAF, NNU, PTB
Shan et al (2016)[20]	362	1110	China	Retrospective cohort	100%	ICD 10 coding, pruritus, ↑TBA, ALT, AST, no other cause of liver dysfunction, pruritus, gallstones, cholecystitis, or cirrhosis	8	2013-2015	TBA, ALT, AST, LDH	Apgar score, BW, CS, GDM, IUFD, MSAF, NNU, PET, placenta praevia, placental abruption, PPH, PPROM, RDS, SGA
Vural Yilmaz et al (2017)[21]	90	90	Turkey	Prospective case-control	0%	Pruritus, ↑TBA, and/or ↑ALT/AST, no rash, no other cause of pruritus	6	2014-2016	TBA, ALT, AST, FBC, red cell distribution width	Apgar score, BW, GA delivery, BW, MSAF, NNU, SGA
Wikström Shemer et al (2013)[22]	333	25 537	Sweden	Retrospective cohort	0%	Pruritus, ↑TBA, and/or ↑ALT/AST, no other cause for pruritus	8	2002-2006	TBA, ALT, AST, bilirubin	BW, CS, fetal asphyxia, GDM, IOL, IUFD, PET, PPH, PTB
Wong et al (2008)[23]	151	602	Republic of Ireland	Retrospective case-control	9·3% ICP 5·2% controls	Pruritus, ↑TBA ± LFTs, no other cause for raised TBA	6	2004-2006	TBA	Apgar score, BW centile, GA delivery, IOL, NNU, NRHRM, PTB
Zhang et al (2014)[24]	40	42	China	Prospective case-control	0%	Pruritus, post-partum normalisation, ↑TBA,	4	2011-2012	TBA, ALT, AST, bilirubin, ALP, lipid	Apgar score, BW, fetal distress, GA delivery, IUFD, MSAF, SPTB

						ALT/AST, no other cause of pruritus, autoimmune disorders, hepatitis viral infection, moderate to severe alcohol intake, biliary obstruction, or the use of drugs that can precipitate cholestasis			profile, serum cytokines	

Data quality was determined by the Newcastle-Ottawa case-control scale at study-level. alb: albumin, ALP: Alkaline phosphatase, ALT: alanine transaminase, APH: antepartum haemorrhage, AST: aspartate aminotransferase, BE: base excess, BP: blood pressure, BW: birthweight, CS: caesarean section, EBL: estimated blood loss, EPO: erythropoietin, FBC: full blood count, GA delivery: gestational age of delivery, GDM: gestational diabetes mellitus, GGT: Gamma-glutamyl transferase, Hb: haemoglobin, ICD: International Classification of Disease, ICH: intra-cerebral haemorrhage, IOL: induction of labour, IPTB: iatrogenic preterm birth, IUFD: intrauterine fetal death, IUGR: intrauterine growth restriction, LBW: low birthweight, LDH: lactate dehydrogenase, LFTs: liver function tests, LGA: large for gestational age, MOD: mode of delivery, MS: meconium-staining, MSAF: meconium-staining of the amniotic fluid, n/a: not available, NND: neonatal death, NNU: neonatal unit admission, NRHRM: non-reassuring heart rate monitoring, pCO_2: partial pressure of carbon dioxide, PCR: protein creatinine ratio, PET: pre-eclampsia, pO_2: partial pressure of oxygen, PPH: post-partum haemorrhage, PPROM: preterm prelabour rupture of membranes, PTB: preterm birth, RDS: respiratory distress syndrome, Rh: Rhesus group, SGA: small for gestational age, SPTB: spontaneous preterm birth, TBA: serum total bile acids, UC: umbilical cord, US: ultrasound

Table 3. Studies used for individual patient data analysis

	Number of cases	Country	Design	Definition of ICP used	Data quality (NHLBI)	Data collection period	Major predictors reported	Method of bile acid measurement	Outcome measures reported
Bacq et al (2017)[25]	82	France	Retrospective cohort	Gestational pruritus, ↑TBA or ALT on two occasions, no other liver disorder, or dermatoses of pregnancy	8	1999-2013	Pruritus severity, TBA, ALT, AST, bilirubin, GGT, ALP, ABCB4 mutations, PT	Enzymatic colorimetric (Enzabile, biostat)	↓ biochemical markers, CS, GA delivery, IOL, PTB
Brouwers et al (2015)[26]	216	Netherlands	Retrospective cohort	Gestational pruritus, ↑TBA	8	2005-2012	TBA, ALT, AST, bilirubin, GGT, ALP, LDH	Enzymatic colorimetric (Sentinel)	Apgar score, BW, fetal asphyxia, IOL, MOD, MSAF, NNU, perinatal death, PPH, PTB, UC arterial pH, UC blood TBA
Castaño et al (2006)[3]	44	Argentina	Prospective case-control	Pruritus, ↑TBA, and ↑ALT/AST after 20/40, no other autoimmune disease, moderate-severe alcohol intake, HIV infection, Hepatitis A, B, or C infection, skin disease, or biliary obstruction, post-partum normalisation	8	2004-2005	Pruritus severity, TBA, individual BA, ALT, AST, bilirubin, GGT, ALP	Capillary electrophoresis and HPLC	Apgar score, BW, GA delivery, SGA
Chappell et al (2012)[27]	128	UK	Randomised controlled trial	Gestational pruritus, ↑TBA or ALT, no other cause of pruritus or liver dysfunction (except concurrent hepatitis C or cholelithiasis)	8	2008-2011	Pruritus severity, TBA, ALT, AST, bilirubin, GGT	Clinical laboratory	Apgar score, BW, EBL, GA delivery, IOL, IUFD, MOD, MSAF, neonatal convulsions, jaundice or ventilation, NNU, PTB, UC arterial pH, UC venous pH
Chen et al (2013)[28]	38	China	Prospective case-control	Pruritus after 28/40, ↑TBA, and ↑ALT/AST, no chronic liver disease or symptomatic cholelithiasis, post-partum normalisation	7	2007-2008	TBA, ALT AST, bilirubin, GGT, ALP	HPLC-MS/MS	GA delivery, IUFD, MSAF, NRHRM, SPTB
Cui et al (2018)[4]	42	China	Prospective case-control	As per RCOG Guideline 2nd edition: i.e. gestational pruritus, ↑TBA and/or ↑ALT/AST, postpartum resolution, no other cause of itching or liver dysfunction[5]	7	2012-2015	TBA, individual BA, ALT, AST, bilirubin, GGT, ALP	Clinical laboratory	BW, CS, EBL, GA delivery, MSAF

Estiú et al (2017)[29]	400	Argentina	Prospective cohort	Pruritus, ↑TBA, no other cause for pruritus, or hepatic disease	8	2009-2013	TBA	Enzymatic colorimetric (Randox)	Apgar score, BW, fetal asphyxia, GA delivery, IUFD, LGA, meconium aspiration syndrome, MSAF, NNU, NRHRM, perinatal death, placental abruption, PTB, SGA
Furrer et al (2016)[6]	344	Switzerland	Retrospective case-control	Database coding	7	2004-2014	TBA	Clinical laboratory	Apgar score, blood transfusion, BW, EBL, GA delivery, Hb pre- and post- delivery, IOL, MOD, MSAF, NNU, perinatal death, UC arterial pH
Geenes et al (2014)[8]	708	UK	Prospective population-based cohort	Pruritus without a rash, severe ICP: TBA≥ 40µmol/L	8	2010-2011	TBA, ALT, AST, bilirubin, GGT	Clinical laboratory	Apgar score, BW, BW centile, GA delivery, IPTB, IUFD, MOD, MSAF, NNU, SPTB
Glantz et al (2007)[30]	40	Sweden	Prospective cohort	Gestational pruritus without dermatological cause, ↑TBA	8	1999-2002	Pruritus severity, TBA, urine BA, ALT, bilirubin, urine steroids	Clinical laboratory	Pruritus severity. Additional outcome measures reported in Glantz et al 2005: fetal asphyxia, EBL, IOL, MSAF, MS placenta and membranes, PTB, SPTB
Grymowicz et al (2016)[10]	157	Poland	Prospective case-control	Pruritus onset after 20/40, ↑TBA or ALT/AST, no other cause of pruritus, liver disease, or dermatological disease	8	2005-2006	Pruritus severity, TBA, ALT, AST, bilirubin	Enzymatic colorimetric (Enzabile Biostat)	Apgar score, BW, congenital anomalies, fetal asphyxia, GA delivery, ICH, IOL, MOD, MSAF, ↑neonatal bilirubin, NNU, RDS
Günaydin et al (2017)[31]	38	Turkey	Retrospective cohort	ICD codes, ↑TBA	6	2015	↑TBA, ALT, AST, bilirubin, GGT, LDH, coagulation profile	Clinical laboratory	Anaesthesia, Apgar score, BW, GA delivery, IUFD, MOD, SPTB
Kawakita et al (2015)[32]	233	USA	Retrospective cohort	Pruritus, ↑TBA, no rash	8	2009-2014	TBA, AST, ALT	Enzymatic colorimetric (Enzabile)	Blood transfusion, BW, chorioamnionitis, endometritis, GA delivery, IOL, IUFD, IUGR, MOD, MSAF, ↑neonatal bilirubin, NNU, NRHRM, oligohydramnios, placental abruption, PPH, PPROM, RDS, SPTB, TTN

Kebapcilar et al (2010)[13]	40	Turkey	Prospective case-control	Pruritus onset after 20/40, ↑TBA, no other liver disease, or skin disease	7	2008-2009	TBA, ALT, AST, ALP, lipid, and coagulation profiles	Clinical laboratory	Apgar score, GA delivery, IUFD, US doppler indices
Kenyon et al (2002)[33]	73	UK	Prospective cohort	Pruritus, ↑TBA or ↑ALT/AST/GGT, no other liver disease, postnatal resolution	8	1999-2001	Pruritus	Enzymatic colorimetric (Biochemical Enterprise)	APH, IOL, IPTB, IUFD, MOD, MSAF, NNU, NRHRM, PPH, SGA, SPTB
Kohari et al (2017)[34]	857	USA	Retrospective cohort	Pruritus, ↑TBA	7	2005-2013	TBA, ALT, AST	Clinical laboratory	Apgar score, BW, chorioamnionitis, GA delivery, IOL, IUFD, MOD, MSAF, neonatal antibiotic use, NNU, NRHRM, PPROM, RDS, SGA, UC arterial pH
Kondrackiene et al (2007)[35]	66	Lithuania	Retrospective cohort	Pruritus onset in 2nd/3rd trimesters, ↑TBA, no other liver disease, skin disease, allergic disorder, symptomatic cholelithiasis, or ongoing viral infection affecting the liver	7	1999-2002	Pruritus severity, TBA, LFTs	Clinical laboratory	Apgar score, BW, fetal asphyxia, GA delivery, MOD, SPTB
Kowalska-Kańka et al (2013)[14]	41	Poland	Case-control	Pruritus, ↑TBA, and ↑ALT/AST, no other cause of pruritus, liver or biliary tract disorder	7	2009-2011	Pruritus severity, TBA, ALT, AST, bilirubin, EPO, FBC, coagulation profile	Enzymatic colorimetric (Randox)	Apgar score, BW, GA delivery, NRHRM, UC arterial pH, pCO_2, pO_2, and BE, US indices
Lee et al (2008)[36]	117	USA	Retrospective cohort	ICP notes coding, pruritus, ↑TBA, no rash	7	2000-2007	TBA, ALT, AST, bilirubin	Clinical laboratory	Apgar score, BW, chorioamnionitis, EBL, IOL, IUFD, MOD, MSAF, NRHRM, PPH, SGA, SPTB, UC arterial pH
Marathe et al (2017)[37]	344	Australia	Retrospective cohort	Pruritus, ↑TBA	8	2000-2010	TBA	Enzymatic colorimetric (Enzabile, Randox)	Apgar score, BW, feeding difficulties, GA delivery, IUFD, length of hospital stay, LGA, mechanical ventilation, MOD, MSAF, neonatal hypoglycaemia, neonatal jaundice, NNU, RDS, SGA, SPTB, TTN, UC arterial pH

Raz et al (2015)[17]	78	Israel	Retrospective cohort	Pruritus, ↑TBA, and LFTs, no other viral infection affecting the liver, liver disease, or biliary disease	8	2008-2014	TBA, LFTs, FBC, proteinuria, PCR, BP	Enzymatic colorimetric (Diazyme)	Apgar score, BW, BW centile, GA delivery, IOL, IUFD, MOD, MSAF, neonatal complications, NRHRM, PET, PTB, UC blood pH
Roncaglia et al (2002)[18]	207	Italy	Prospective cohort	Pruritus onset in 2nd/3rd trimesters, post-partum normalisation, ↑TBA or ↑ALT/AST, no other cause of pruritus	8	1989-1997	Pruritus severity, TBA, ALT, AST, bilirubin, BP	Clinical laboratory	Apgar score, GA delivery, IOL, IUFD, meconium aspiration, MOD, MSAF, oligohydramnios, PTB, SGA, UC arterial pH
Rook et al (2012)[38]	101	USA	Retrospective cohort	ICD9 coding, pruritus, onset in 2nd /3rd trimesters, no other cause for pruritus, chronic liver disease, acute fatty liver of pregnancy, or HELLP syndrome	8	2005-2009	TBA, individual BA, ALT, AST, bilirubin, ALP, alb	Clinical laboratory	Apgar score, BW, congenital anomalies, fetal distress, GA delivery, IOL, meconium aspiration, MOD, MSAF, ↑neonatal bilirubin, neonatal pneumonia, RDS, sepsis
Shan et al (2016)[20]	362	China	Retrospective cohort	ICD 10 coding, pruritus, ↑TBA, ALT, AST, no other cause of liver dysfunction, pruritus, gallstones, cholecystitis, or cirrhosis	9	2013-2015	TBA, ALT, AST, LDH	Clinical laboratory	Apgar score, BW, CS, GDM, IUFD, MSAF, NNU, PET, placenta praevia, placental abruption, PPH, PPROM, RDS, SGA
Yayla Abide et al (2017)[39]	91	Turkey	Retrospective case-control	Pruritus, ↑TBA, no other cause, no other liver disease	9	2013-2016	TBA, ALT, AST, GGT, ALP, LDH, FBC, fibrinogen	Enzymatic colorimetric (Diazyme)	Apgar score, BW, CS, GA delivery, IUFD, MSAF, NND, NNU
Imperial	313	UK	Prospective cohort	Gestational pruritus, ↑TBA, no other cause for pruritus, or hepatic dysfunction	N/a	2006–2010, 2011-2017*	Pruritus severity, TBA, ALT, AST, bilirubin	Clinical laboratory	Apgar score, BW, EBL, GA delivery, GDM, IOL, IUFD, MOD, MSAF, NND, NNU, NRHRM, PTB, SPTB, UC arterial pH
GSTT	109	UK	Prospective cohort	Gestational pruritus, ↑TBA, no other cause for pruritus, or hepatic dysfunction	N/a	2011-2017*	Pruritus severity, TBA, ALT, AST, bilirubin	Clinical laboratory	Apgar score, BW, EBL, GA delivery, GDM, IOL, IUFD, MOD, MSAF, NND, NNU, NRHRM, PTB, SPTB, UC arterial pH

Data quality was determined by the NHLBI Quality Assessment Tool for Case Series Studies. *Participants that gave birth between 2010-2011 were not included to avoid duplication with the recruitment period for Geenes *et al*., 2014[8]. alb: albumin, ALP: Alkaline phosphatase, ALT: alanine transaminase, AST: aspartate aminotransferase, BE: base excess, BP: blood pressure, BW: birthweight, CS: caesarean section, EBL: estimated blood loss, EPO: erythropoietin, FBC: full blood count, GA delivery: gestational age of delivery, GDM: gestational diabetes mellitus, GGT: Gamma-glutamyl transferase, Hb: haemoglobin, HELLP: haemolysis, elevated liver enzymes, low platelets, HPLC: high performance liquid chromatography, ICD: International Classification of Disease, ICH: intra-cerebral haemorrhage, IOL: induction of labour, IPTB: iatrogenic preterm birth, IUFD: intrauterine fetal death, IUGR: intrauterine growth restriction,

LDH: lactate dehydrogenase, LFTs: liver function tests, LGA: large for gestational age, MOD: mode of delivery, MS: meconium-staining, MSAF: meconium-staining of the amniotic fluid, N/a: not applicable; NND: neonatal death, NNU: neonatal unit admission, NOS: Newcastle-Ottawa score, NRHRM: non-reassuring heart rate monitoring, pCO_2: partial pressure of carbon dioxide, pO_2: partial pressure of oxygen, PPH: post-partum haemorrhage, PPROM: preterm prelabour rupture of membranes, PTB: preterm birth, RCT: randomised controlled trial, RDS: respiratory distress syndrome, SGA: small for gestational age, SPTB: spontaneous preterm birth, TBA: serum total bile acids, TTN: transient tachypnoea of the newborn, UC: umbilical cord, US: ultrasound

Table 4. Studies not included in individual patient data analysis for which data were requested

	Number of cases	Country	Design	Included in aggregate data meta-analysis?	Data collection period	Reason not included in IPD analysis
Al Shobaili et al (2011)[1]	76	Saudi Arabia	Prospective cohort	Yes	2008-2010	No reply
Ataalla et al (2016)[2]	98	Egypt	Prospective case-control	Yes	2011-2013	No reply
Garcia-Flores et al (2015)[7]	47	Spain	Prospective cohort	Yes	2012-2014	Data not available
Glantz et al (2004)[9]	505	Sweden	Prospective cohort	Yes	1999-2002	Data not available
Heinonen and Kirkinen (1999)[11]	91	Finland	Prospective population cohort	Yes	1990-1996	Data not available
Herrera et al (2018)[12]	487	USA	Retrospective cohort	Yes	2005-2015	No reply
Liu et al (2016)[15]	1319	China	Retrospective population cohort	Yes	2006-2014	Data not available
Oztas et al (2015)[16]	117	Turkey	Prospective case-control	Yes	2012-2014	No reply
Sargin Oruç et al (2014)[19]	57	Turkey	Prospective case-control	Yes	2012	Duplicate dataset
Vural Yilmaz et al (2017)[21]	90	Turkey	Prospective case-control	Yes	2014-2016	No reply
Wikström Shemer et al (2013)[22]	333	Sweden	Retrospective cohort	Yes	2002-2006	Data not available
Wong et al (2008)[23]	151	Republic of Ireland	Retrospective cohort	Yes	2004-2006	No reply
Zhang et al (2014)[24]	40	China	Prospective case-control	Yes	2011-2012	No reply
Binder et al (2006)[40]	78	Czech Republic	Randomised controlled trial	No	1999-2004	Data not available
Bolukbas et al (2017)[41]	59	Turkey	Retrospective cohort	No	2007-2014	No reply
Chen et al (2013)[42]	106	China	Retrospective cohort	No	1990-2011	No reply
Erkenekli et al (2015)[43]	103	Turkey	Retrospective cohort	No	2008-2013	No reply
Friberg et al (2016)[44]	113	Denmark	Retrospective cohort	No	2006-2011	No reply
Jin et al (2015)[45]	371	China	Retrospective cohort	No	1993-2014	No reply
Joutsiniemi et al (2015)[46]	103	Turkey	Prospective cohort	No	2008-2013	Data not available
Koroglu et al (2017)	40	Turkey	Prospective case-control	No	2016-2017	Data not available
Labbe et al (2018)[47]	138	France	Retrospective cohort	No	2008-2014	Data not available
Lin et al (2017)[48]	407	China	Retrospective cohort	No	2014-2016	No reply
Madazli et al (2015)[49]	89	Turkey	Retrospective cohort	No	2003-2013	No reply
Oztekin et al (2009)[50]	187	Turkey	Retrospective cohort	No	2004-2008	No reply
Pata et al (2011)[51]	32	Turkey	Cohort	No	2006-2010	No reply
Riikonen et al (2000)[52]	39	Finland	Randomised controlled trial	No	≤1998	Data not available
Zecca et al (2008)[53]	77	Italy	Retrospective cohort	No	2000-2004	No reply
Zhang et al (2015)[54]	120	China	Randomised controlled trial	No	2009-2011	No reply

IPD: individual patient data, UK: United Kingdom; USA: United States of America

Table 5. Demographic and pregnancy characteristics of women included in the systematic review and IPD analysis

	Cases (data set 1 - study level)	Controls (data set 1 – study level)	Cases (data set 2 – IPD level)	Comparison 1 data set 1 cases vs controls	Comparison 2 data set 1 cases vs data set 2 cases
Maternal age (years) mean (SD)	30·0 (6·1) N=3237	30·2 (5·5) N=34 863	29·7 (6·0) N=5228	Mean diff -0·2 (-0·4 to 0·0)	Mean diff 0·3 (0·0 to 0·6)
BMI (kg/m^2) mean (SD)	25·4 (8·1) N=2768	23·8 (4·7) N=7796	24·8 (5·2) N=2464	Mean diff 1·6 (1·3 to 1·9)	Mean diff 0·6 (0·2 to 1·0)
Ethnicity n (%):					
White	1054 (38·9)	2035 (46·8)	3488 (73·6)	OR 0·7 (0·7 to 0·8)	OR 0·2 (0·1 to 0·3)
Asian	1483 (54·7)	2033 (46·7)	867 (18·3)	OR 1·4 (1·2 to 1·9)	OR 5·4 (4·9 to 6·0)
Black	24 (0·9)	131 (3·0)	130 (2·7)	OR 0·3 (0·2 to 0·4)	OR 0·3 (0·2 to 0·5)
Mixed/Other	151 (5·6) N=2712	151 (3·5) N=4350	255 (5·4) N=4740	OR 1·6 (1·3 to 2·1)	OR 1·0 (0·8 to 1·3)
Nulliparity n (%)	1499 (55·8) N=2688	20 140 (59·0) N=34 152	1874 (36·9) N=5076	OR 0·9 (0·8 to 0·9)	OR 2·2 (2·0 to 2·4)
Multifetal pregnancy n (%)	760 (17·1) N=4453	3489 (2·8) N=125 417	752* (14.·3) N=5270	OR 7·2 (6·6 to 7·8)	OR 1·2 (1·1 to 1·4)
Pre-eclampsia n (%)	228 (12·2) N=1876	3385 (3·4) N=94 386	312 (7·1) N=4384	OR 3·7 (3·2 to 4·3)	OR 1·8 (1·5 to 2·2)
Gestational diabetes mellitus n (%)	239 (13·2) N=1806	5571 (5·9) N=94384	522 (14·7) N=3545	OR 2·4 (2·1 to 2·8)	OR 0·9 (0·7 to 1·0)

*747=twin, 5=triplet pregnancies. Comparisons show mean difference or odds ratio with 95% confidence intervals. BMI: body mass index, CI: confidence interval, Mean diff: mean difference, n: number of women with characteristic, N: number of women reporting these data, OR: odds ratio, SD: standard deviation.

Table 6. Description of ICP disease

	Cases (data set 1 - study level)	Controls (data set 1 – study level)	Cases (data set 2 – IPD level)	Comparison 1 data set 1 cases vs controls	Comparison 2 data set 2 cases vs data set 1 cases
Total bile acids (µmol/L) mean (SD)	45·4 (42·6) N=2799	6·0 (4·1) N=555	50·1 (50·2) N=5159	Mean diff 39·4 (37·8 to 41·0)	Mean diff 4·7 (2·6 to 6·8)
ALT (iU/L) mean (SD)	147·6 (165·5) N=2252	25·9 (21·8) N=718	157·7 (171·7) N=4477	Mean diff 121·7 (109·6 to 133·8)	Mean diff 10·1 (1·5 to 18·7)
AST (iU/L) mean (SD)	105·0 (108·6) N=2146	20·9 (11·7) N=518	105·7 (114·8) N=3845	Mean diff 84·1 (74·7 to 93·5)	Mean diff 0·7 (-5·2 to 6·6)
Bilirubin (µmol/L) mean (SD)	12·9 (7·5) N=1548	7·1 (3·8) N=532	14·7 (12·5) N=2962	Mean diff 5·8 (5·1 to 6·5)	Mean diff -1·8 (1·1 to 2·5)
UDCA treatment n (%)	2112 (67·9) N=3112	n/a	3304 (65·6) N=5034	n/a	OR 0·9 (0·8 to 1·0)
Bile acids taken fasting n (%)	934 (42·6) N=2190	505 (100·0) N=505	1726 (32·8) N=5270	OR 0·0 (0·0 to 0·0)	OR 0·7 (0·6 to 0·7)

Comparisons show mean difference or odds ratio, and 95% confidence interval. ALT: alanine aminotransferase, AST: aspartate aminotransferase, CI: confidence interval, IPD: individual patient data, mean diff: mean difference, n: number of women with characteristic, N: number of women reporting these data, OR: odds ratio, SD: standard deviation, UDCA: ursodeoxycholic acid.

Table 7. Summary table of results – aggregate data case-control comparisons

Perinatal outcome	Number of cases	Number of controls	Aggregate data comparison – ICP vs control	
			Weighted mean difference / Odds ratio (95% CI)	Tau-squared (p value)
Stillbirth	45/5427	519/164601	1·46 (0·73 to 2·89)	0·81 (0·0016)
Preterm birth	1270/4289	10956/160822	3·54 (2·72 to 4·62)	0·18 (<0·0001)
Spontaneous preterm birth	412/3080	4892/121208	3·47 (3·06 to 3·95)	0·55 (<0·0001)
Iatrogenic preterm birth	305/2576	2440/121011	3·65 (1·94 to 6·85)	0·61 (<0·0001)
Gestation at delivery (weeks)	2276 (37.1)	7007 (37.8)	-1·48 (-1·96 to -1·01)	0·83 (<0·0001)
Caesarean section	942/2846	12312/69235	1·16 (0·92 to 1·48)	0·14 (<0·0001)
Meconium stained amniotic fluid	360/1910	2401/22260	2·60 (1·62 to 4·16)	0·51 (<0·0001)
Apgar <7 at 5 minutes	48/1766	571/31943	1·41 (0·95 to 2·10)	0·00 (0·47)
Neonatal unit admission	517/2175	3367/26005	2·12 (1·48 to 3·03)	0·24 (<0·0001)
Birthweight centile	818	2703	0·60 (-6·21 to 7·41)	35·18 (0·0051)

Comparisons using random effects model. CI: confidence interval. Meta-regression was performed to assess the effect of confounders on outcomes presented; assessments of study quality (determined by Newcastle-Ottawa score), the inclusion of multifetal pregnancies versus singleton pregnancies, and use of uncomplicated pregnancies as controls versus pregnant women with pruritus but normal total bile acids did not significantly contribute to the heterogeneity of the results (p>0.05 throughout), with the exception of the effect of study quality on proportions of pregnancies with meconium-staining of the amniotic fluid (p=0.0108).

Table 8. Summary of IPD associations between serum biochemistry and adverse perinatal outcome for multifetal pregnancies

Perinatal outcome	Bile acids ROC AUC (95% CI) (n/N)	ALT ROC AUC (95% CI) (n/N)	AST ROC AUC (95% CI) (n/N)	Bilirubin ROC AUC (95% CI) (n/N)
Stillbirth	0·48 (0·34 to 0·63) (14/748)	0·47 (0·30 to 0·65) (14/642)	0·52 (0·35 to 0·68) (14/608)	0·49 (0·00 to 1·00) (3/253)
Preterm birth	0·57 (0·51 to 0·62) (598/747)	0·59 (0·50 to 0·68) (160/207)	0·61 (0·52 to 0·69) (160/207)	0.48 (0·39 to 0·57) (160/207)
Spontaneous preterm birth	0·55 (0·49 to 0·61) (165/434)	0·55 (0·47 to 0·64) (75/199)	0·57 (0·49 to 0·66) (75/199)	0·37 (0·28 to 0·45) (75/199)
Iatrogenic preterm birth	0·57 (0·51 to 0·62) (120/434)	0·52 (0·43 to 0·61) (77/199)	0·51 (0·43 to 0·60) (77/199)	0·62 (0·54 to 0·70) (77/199)
Meconium stained amniotic fluid	0·59 (0·53 to 0·65) (101/714)	0·51 (0·40 to 0·63) (29/179)	0·48 (0·39 to 0·63) (29/179)	0·40 (0·35 to 0·60) (29/179)
Non-reassuring heart rate monitoring	0·66 (0·56 to 0·76) (42/198)	0·62 (0·48 to 0·76) (23/140)	0·67 (0·54 to 0·79) (23/140)	0·41 (0·27 to 0·55) (23/140)
Apgar <7 at 5 minutes	0·49 (0·39 to 0·59) (30/739)	0·61 (0·40 to 0·81) (11/203)	0·66 (0·48 to 0·85) (11/203)	0·48 (0·26 to 0·70) (11/203)
Umbilical cord arterial blood pH <7·0	0·67 (0·46 to 0·89) (4/221)	0·15 (0·00 to 0·35) (3/87)	0·10 (0·00 to 0·20) (3/87)	0·57 (0·11 to 1·00) (3/87)
Neonatal unit admission	0·55 (0·51 to 0·60) (298/719)	0·49 (0·41 to 0·58) (77/181)	0·50 (0·42 to 0·59) (77/181)	0·65 (0·58 to 0·73) (77/181)
Neonatal death	0·57 (0·29 to 0·85) (6/246)	0·62 (0·43 to 0·82) (4/174)	0·56 (0·44 to 0·68) (4/174)	0·29 (0·03 to 0·55) (4/174)

ALT: alanine aminotransferase, AST: aspartate aminotransferase, CI: confidence interval, n: number of adverse events (counted as maximum 1 per pregnancy), N: number of women for whom data was available, ROC AUC: receiver operating characteristic area under curve.

Table 9. Sensitivity analysis of IPD associations between serum biochemistry and adverse perinatal outcomes for singleton pregnancies excluding unpublished studies

Perinatal outcome	Bile acids ROC AUC (95% CI) (N)	ALT ROC AUC (95% CI) (N)	AST ROC AUC (95% CI) (N)	Bilirubin ROC AUC (95% CI) (N)
Stillbirth	0.95 (0.93 to 0.97) (5/1480)	0.56 (0.26 to 0.86) (5/1480)	0.57 (0.33 to 0.82) (5/1480)	0.79 (0.62 to 0.95) (5/1480)
Preterm birth	0.55 (0.52 to 0.58) (532/1582)	0.53 (0.50 to 0.56) (532/1582)	0.53 (0.50 to 0.56) (532/1582)	0.57 (0.54 to 0.60) (532/1582)
Spontaneous preterm birth	0.61 (0.56 to 0.66) (131/1549)	0.58 (0.52 to 0.63) (131/1549)	0.58 (0.53 to 0.63) (131/1549)	0.56 (0.51 to 0.62) (131/1549)
Iatrogenic preterm birth	0.53 (0.49 to 0.56) (368/1549)	0.52 (0.48 to 0.55) (368/1549)	0.51 (0.48 to 0.54) (368/1549)	0.56 (0.53 to 0.59) (368/1549)
Meconium stained amniotic fluid	0.59 (0.55 to 0.63) (220/1386)	0.59 (0.55 to 0.63) (220/1386)	0.59 (0.55 to 0.63) (220/1386)	0.57 (0.52 to 0.61) (220/1386)
Non-reassuring heart rate monitoring	0.63 (0.58 to 0.68) (157/1164)	0.49 (0.44 to 0.54) (157/1164)	0.54 (0.49 to 0.59) (157/1164)	0.53 (0.48 to 0.58) (157/1164)
Apgar <7 at 5 minutes	0.64 (0.53 to 0.74) (30/1457)	0.47 (0.37 to 0.56) (30/1457)	0.50 (0.41 to 0.60) (30/1457)	0.53 (0.41 to 0.65) (30/1457)
Umbilical cord arterial blood pH <7·0	0.77 (0.57 to 0.98) (3/426)	0.41 (0.00 to 0.87) (3/426)	0.45 (0.00 to 0.92) (3/426)	0.71 (0.24 to 1.00) (3/426)
Neonatal unit admission	0.59 (0.54 to 0.63) (163/1297)	0.58 (0.53 to 0.62) (163/1297)	0.58 (0.53 to 0.63) (163/1297)	0.55 (0.50 to 0.60) (163/1297)
Neonatal death	0.75 (0.42 to 1.00) (4/1133)	0.56 (0.22 to 0.90) (4/1133)	0.59 (0.28 to 0.90) (4/1133)	0.65 (0.47 to 0.82) (4/1133)

ALT: alanine aminotransferase, AST: aspartate aminotransferase, CI: confidence interval, N: number of women for whom data was available, ROC AUC: receiver operating characteristic area under curve

Table 10. National rates of stillbirth from 28 gestational weeks for studies included in the individual patient data analysis

Country of participants	Studies (by author and year)	Number of patients	Recruitment years	National stillbirth rate 2000 (%)	National stillbirth rate 2015 (%)
Argentina	Castaño et al (2006)[3]<br>Estiú et al (2017)[29]	44<br>400	2004-2005<br>2009-2013	0·73	0·46
Australia	Marathe et al (2017)[37]	344	2000-2010	0·34	0·27
China	Chen et al (2013)[28]<br>Cui et al (2018)[4]<br>Shan et al (2016)[20]	38<br>42<br>362	2007-2008<br>2012-2015<br>2013-2015	1·45	0·72
France	Bacq et al (2017)[25]	82	1999-2013	0·55	0·47
Israel	Raz et al (2015)	78	2008-2014	0.48	0.42
Italy	Roncaglia et al (2002)[18]	207	1989-1997	0·40	0·33
Lithuania	Kondrackiene et al (2007)[35]	66	1999-2002	0·58	0·32
Netherlands	Brouwers et al (2012)[26]	216	2005-2012	0·53	0·18
Poland	Grymowicz et al (2016)[10]<br>Kowalska-Kańka et al (2013)[14]	157<br>41	2005-2006<br>2009-2011	0·47	0·23
Sweden	Glantz et al (2007)[30]	40	1999-2002	0·38	0·28
Switzerland	Furrer et al (2016)[6]	344	2004-2014	0·32	0·28
Turkey	Günaydin et al (2017)[31]<br>Kebapcilar et al (2010)[13]<br>Abide et al (2017)	38<br>40<br>91	2015<br>2008-2009<br>2013-2016	1·27	0·70
UK	Chappell et al (2012)[27]<br>Geenes et al (2014)[8]<br>Kenyon et al (2002)[33]<br>unpublished Imperial and GSTT data	128<br>708<br>73<br>313<br>109	2008-2011<br>2010-2011<br>1999-2001<br>2006-2017<br>2006-2017	0·37	0·29
USA	Kawakita et al (2015)[32]<br>Kohari et al (2017)[34]<br>Lee et al (2008)[36]<br>Rook et al (2012)[38]	233<br>857<br>117<br>101	2009-2014<br>2005-2013<br>2000-2007<br>2005-2009	0·31	0·30

National stillbirth rates from Blencowe *et al*, reporting stillbirths from 28 gestational weeks.[55] Comparative analyses from our data were thus limited to the equivalent gestational period.

Figure 1. Forest plots of selected perinatal outcomes from aggregated patient data

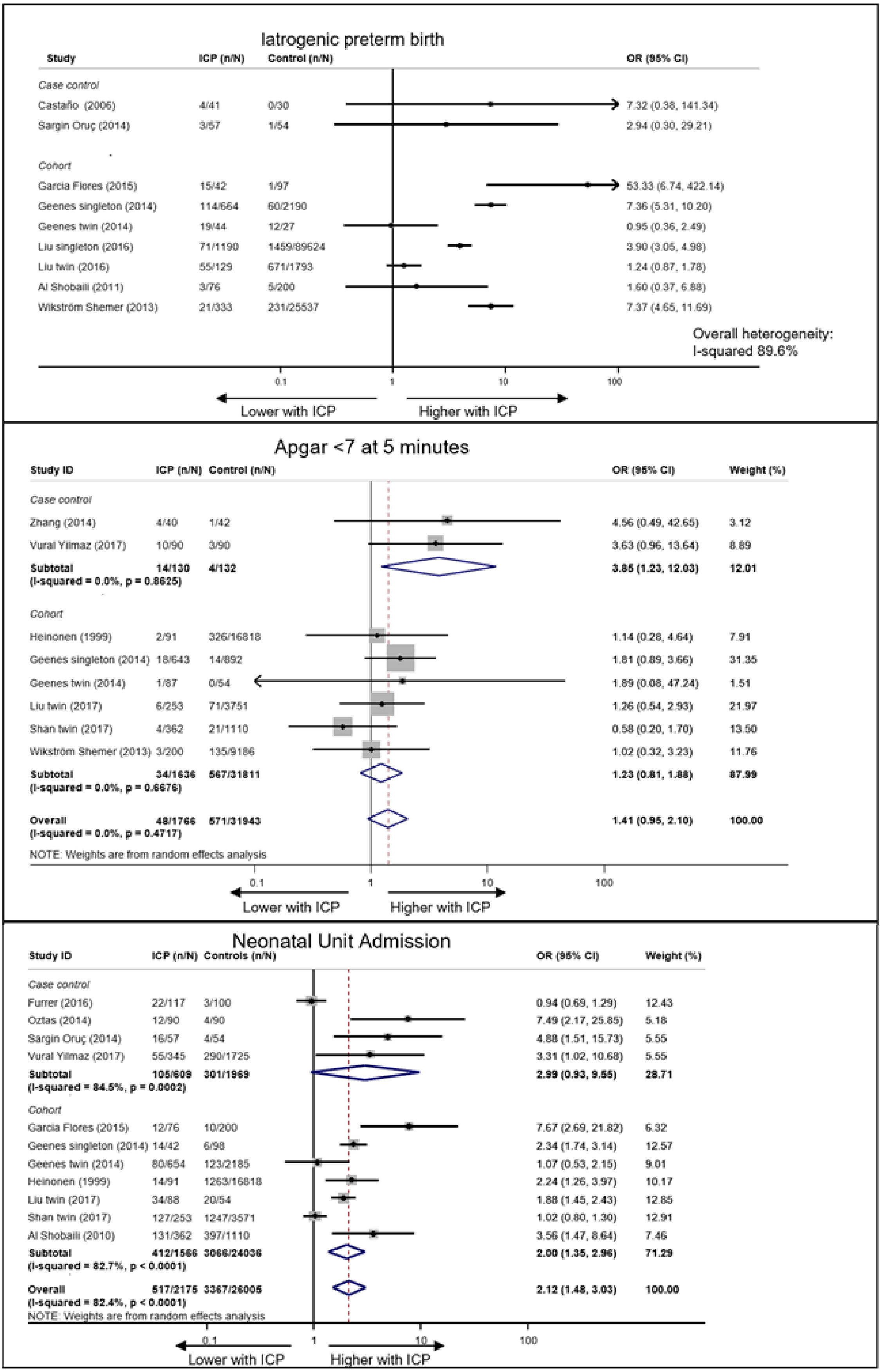

Figure 2. Correction for publication bias in meta-analysis of neonatal unit admission rates in intrahepatic cholestasis of pregnancy

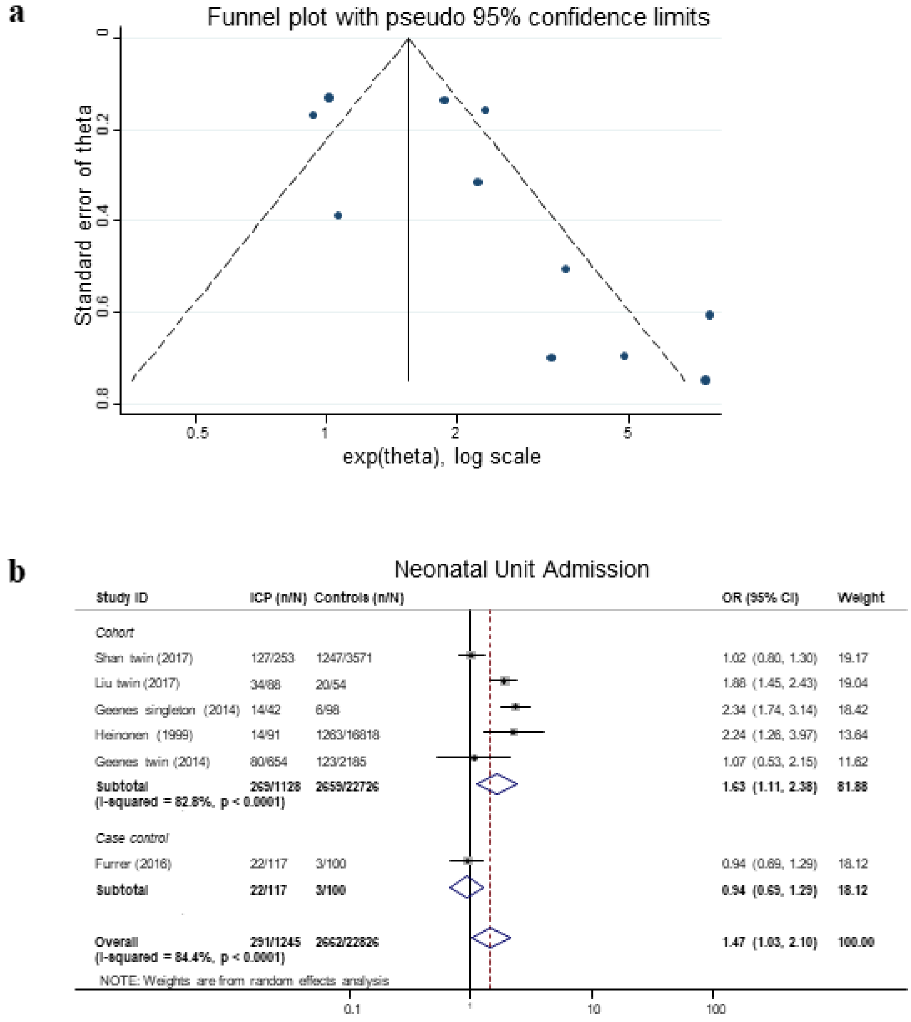

a) Funnel plot of publications included in aggregate data meta-analysis of the association between intrahepatic cholestasis of pregnancy (ICP) and control pregnancies, demonstrating 5 studies likely responsible for the bias in the bottom right of the plot (standard error of theta <0.5)
b) Forest plot of random effects meta-analysis of the effect of ICP on neonatal unit admission excluding the 5 studies demonstrated in (a) as likely to contribute to publication bias

Figure 3. Receiver operating curves for association between stillbirth and serum biochemical markers for multifetal pregnancies

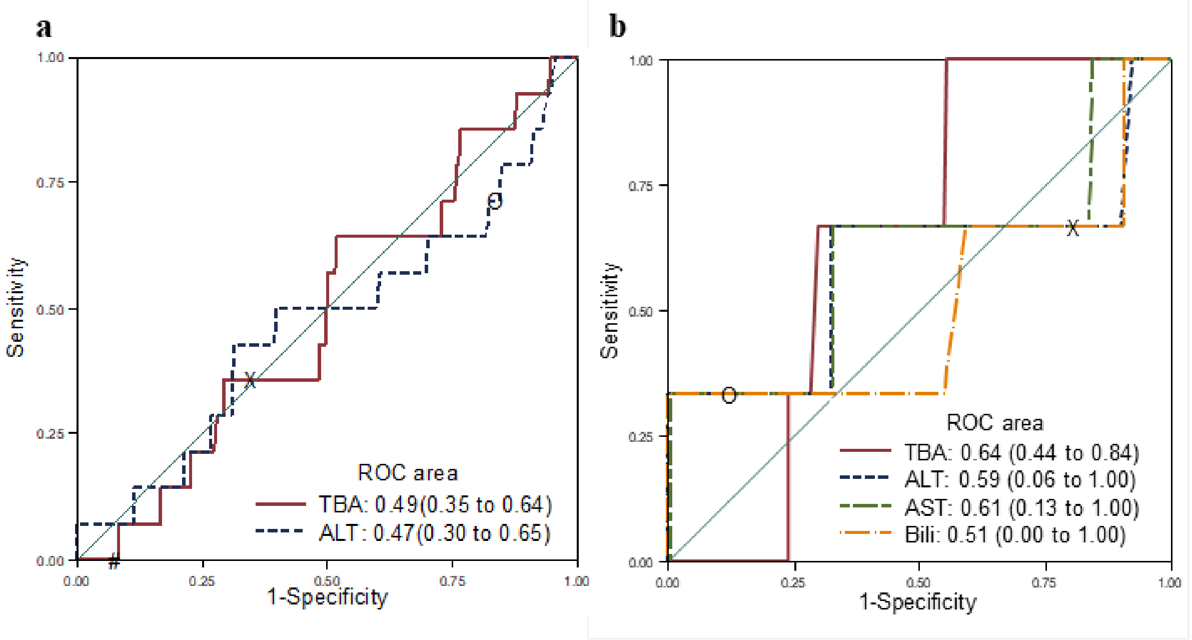

a) Association between stillbirth and peak TBA and ALT concentrations for multifetal pregnancies in a subset of women with both biochemical tests. x: TBA 40µmol/L, #: TBA 100µmol/L, ○: ALT 40IU/L, n=641
b) Association between stillbirth and peak TBA, ALT, AST and Bilirubin concentrations for multifetal pregnancies in a subset of women with both biochemical tests. x: AST 40IU/L, ○: Bilirubin 20µmol/L, n=207

Results are shown for women with results available for all biochemical tests presented in each graph, with ROC area and 95% confidence interval reported for each test. ROC: receiver operating curve.

Figure 4. Receiver operating characteristic curve for the association between total bile acid concentrations and stillbirth for women treated and untreated with ursodeoxycholic acid

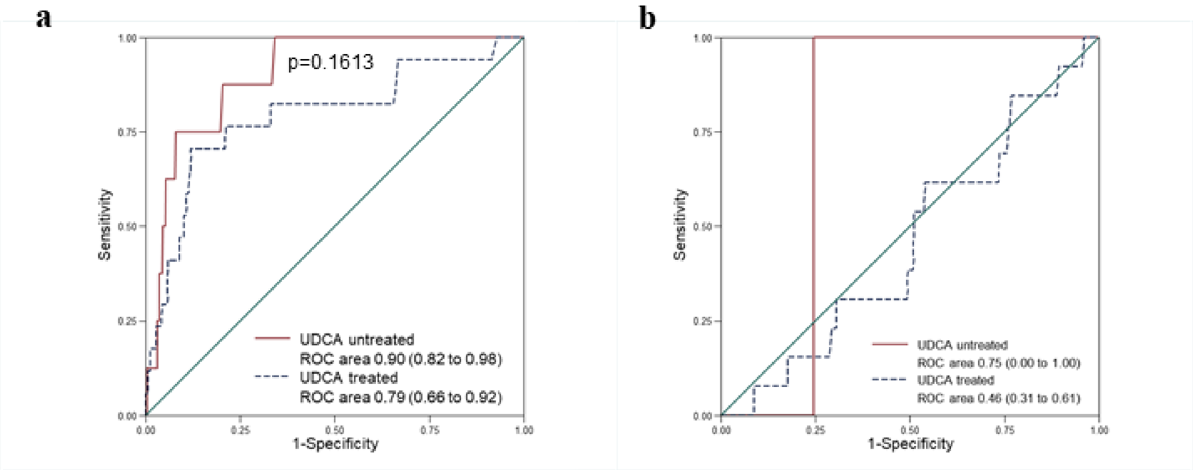

Results show ROC area under curve (95% confidence interval). Comparison between ROC areas shown by p value, determined with Chi-squared test. Statistical comparison not performed for multifetal pregnancies, as only one stillbirth happened in the untreated group. UDCA: ursodeoxycholic acid; ROC: receiver operating characteristic

(a) Singleton pregnancies
(b) Multifetal pregnancies

Figure 5. Proportion and incidence of stillbirths and total number of pregnancies by total bile acid concentrations in multifetal pregnancies with intrahepatic cholestasis of pregnancy

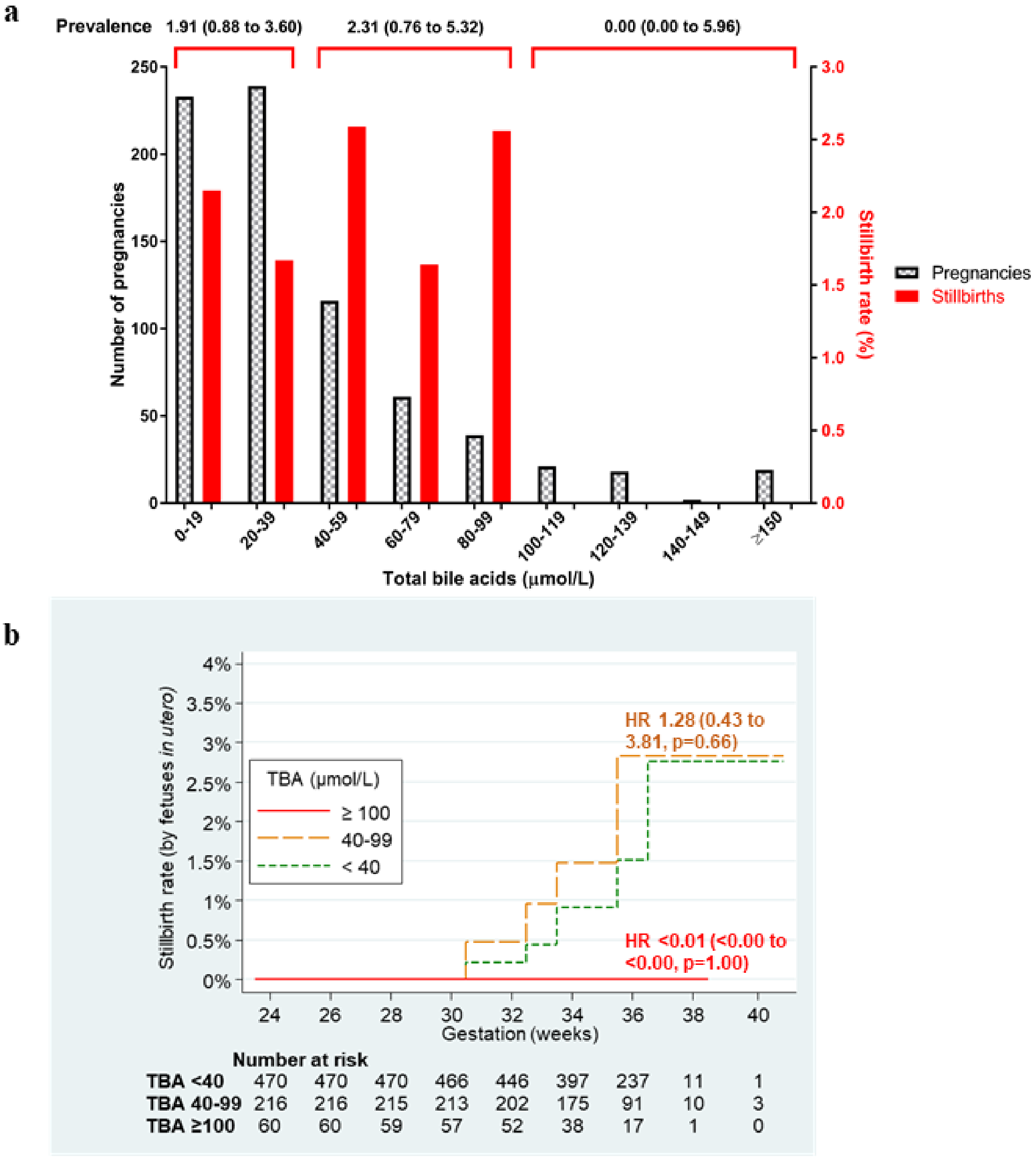

a) Number of women with ICP (black bars) and proportion of stillbirth (red bars) by peak total bile acid category. Stillbirth incidence by total bile acid groups <40µmol/L, 40-99µmol/L and ≥100µmol/L (95% confidence interval)
b) Stillbirth rate by number of fetuses *in utero* until 40 gestational weeks. Data are analysed by completed gestational week categories, with alterations plotted mid-week to reflect uncertainty by individual day of change. Data are not shown from 40 weeks due to the low remaining numbers of fetuses *in utero*. HR: hazard ratio, showing HR of stillbirth compared with women with bile acids <40µmol/L (95% confidence interval)

Figure 6. Stillbirth rate by number of fetuses *in utero* for singleton pregnancies

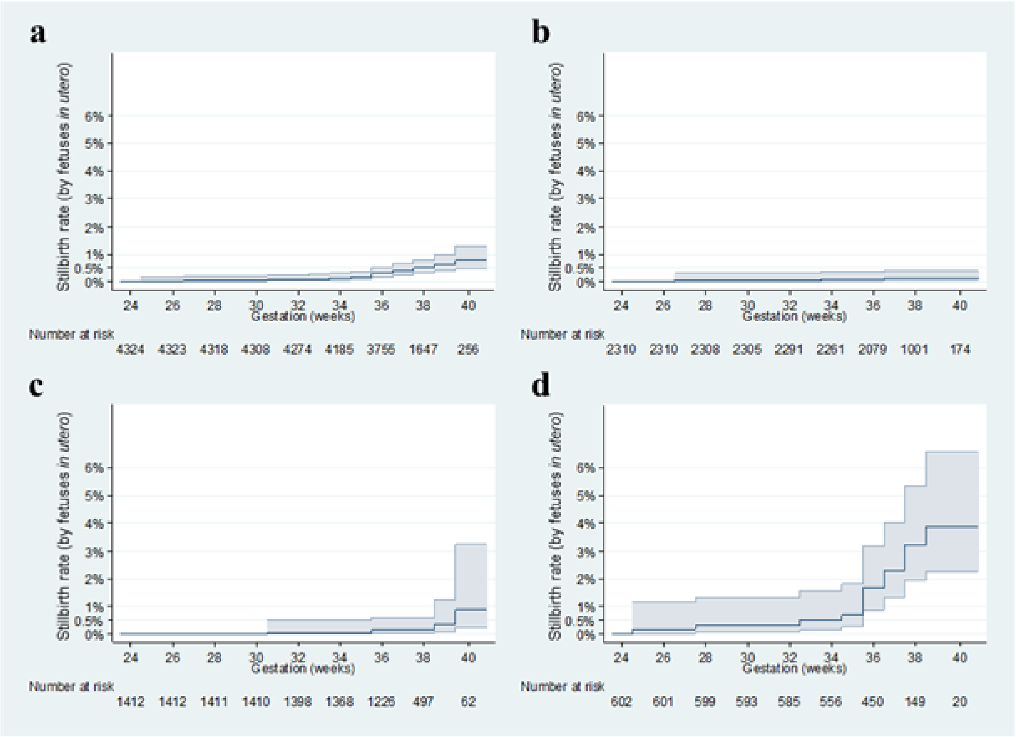

Grey shaded area indicates 95% confidence interval

(a) All participants

(b) Women with total bile acids <40μmol/L

(c) Women with total bile acids 40-99μmol/L

(d) Women with total bile acids ≥100μmol/L

Figure 7. Sensitivity analysis of stillbirth rate by number of fetuses *in utero* for singleton pregnancies

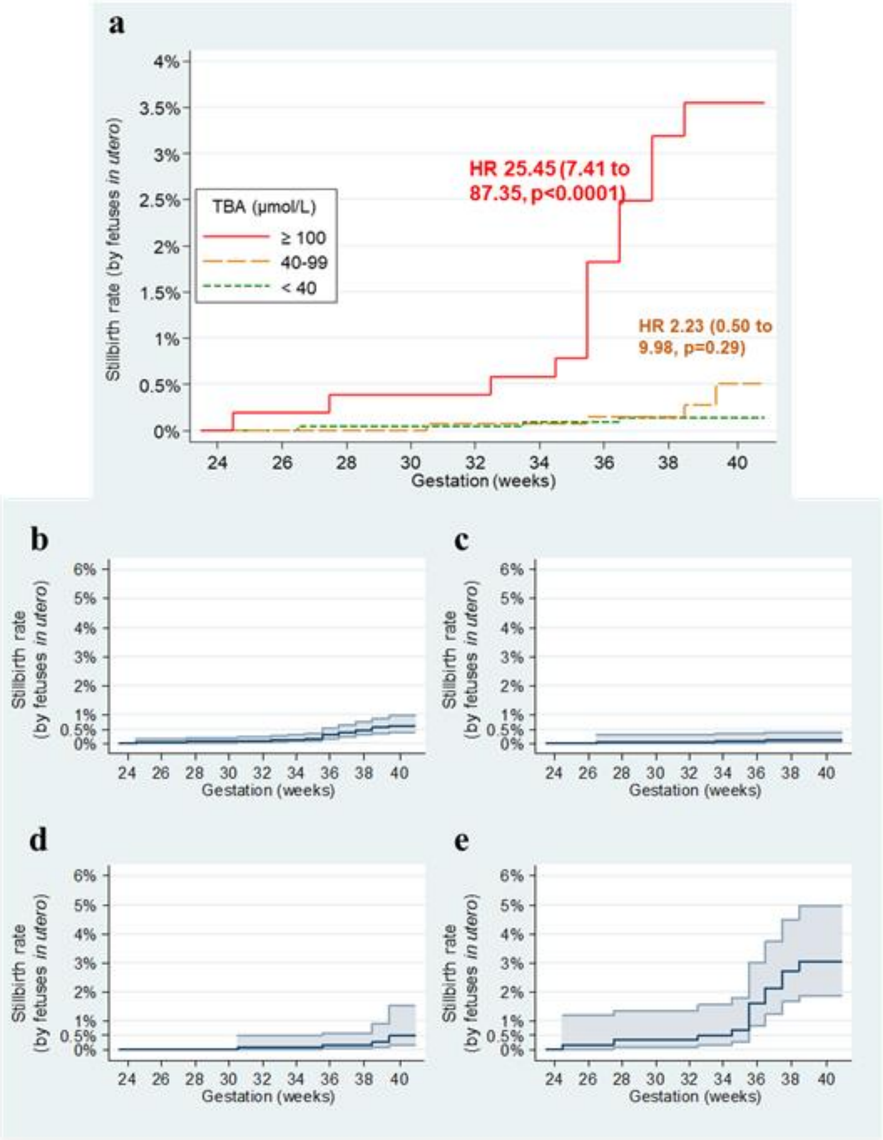

Analysis performed on the assumption that all iatrogenic deliveries would not have been followed by stillbirth before 40 gestational weeks. Results are presented for singleton pregnancies

(a) Stillbirth rate by number of fetuses *in utero* until 40 gestational weeks. Data are analysed by completed gestational week categories, with alterations plotted mid-week to reflect uncertainty by individual day of change. Data are not shown from 40 weeks due to the low remaining numbers of fetuses *in utero*. HR: hazard ratio, showing HR of stillbirth compared with women with bile acids <40µmol/L (95% confidence interval). HR for women with bile acids ≥100µmol/L compared with bile acids 40-99: 5.17 (2.29 to 11.70, p<0.0001)

(b) All participants

(c) Women with total bile acids <40µmol/L

(d) Women with total bile acids 40-99µmol/L

(e) Women with total bile acids ≥100µmol/L

Grey shaded areas indicate 95% confidence intervals

Figure 8. Spontaneous preterm birth rate by number of fetuses *in utero* for singleton pregnancies

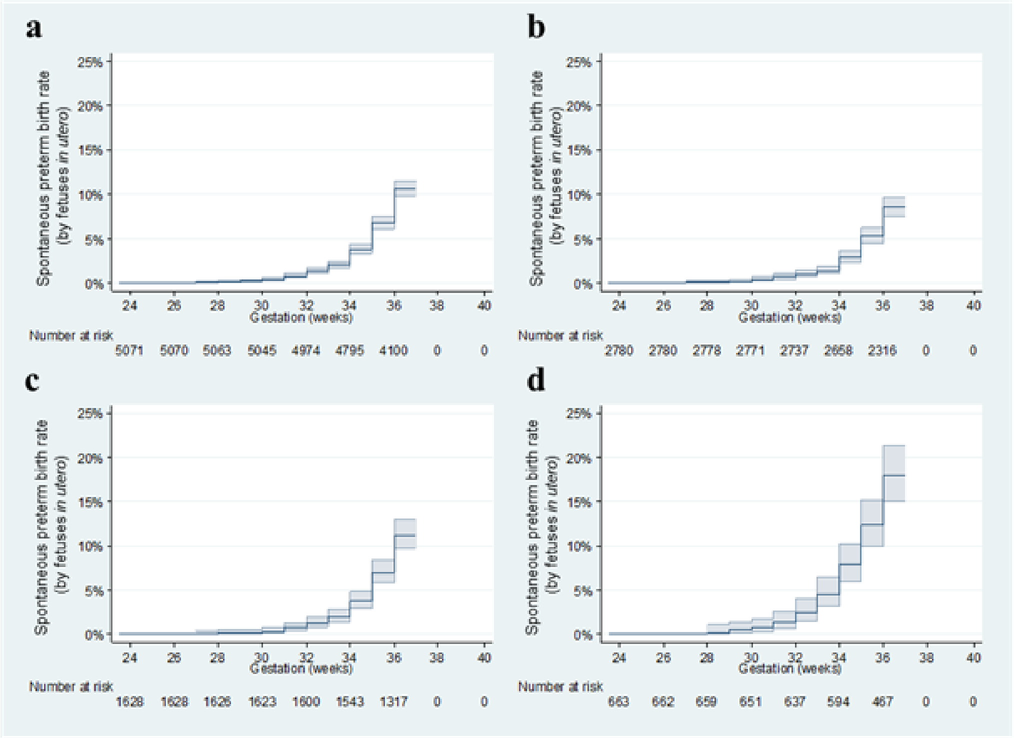

Grey shaded area indicates 95% confidence interval

(a) All participants
(b) Women with total bile acids <40µmol/L
(c) Women with total bile acids 40-99µmol/L
(d) Women with total bile acids ≥100µmol/L

Figure 9. Proportion and incidence of preterm births and total number of pregnancies by total bile acid concentrations in multifetal pregnancies with intrahepatic cholestasis of pregnancy

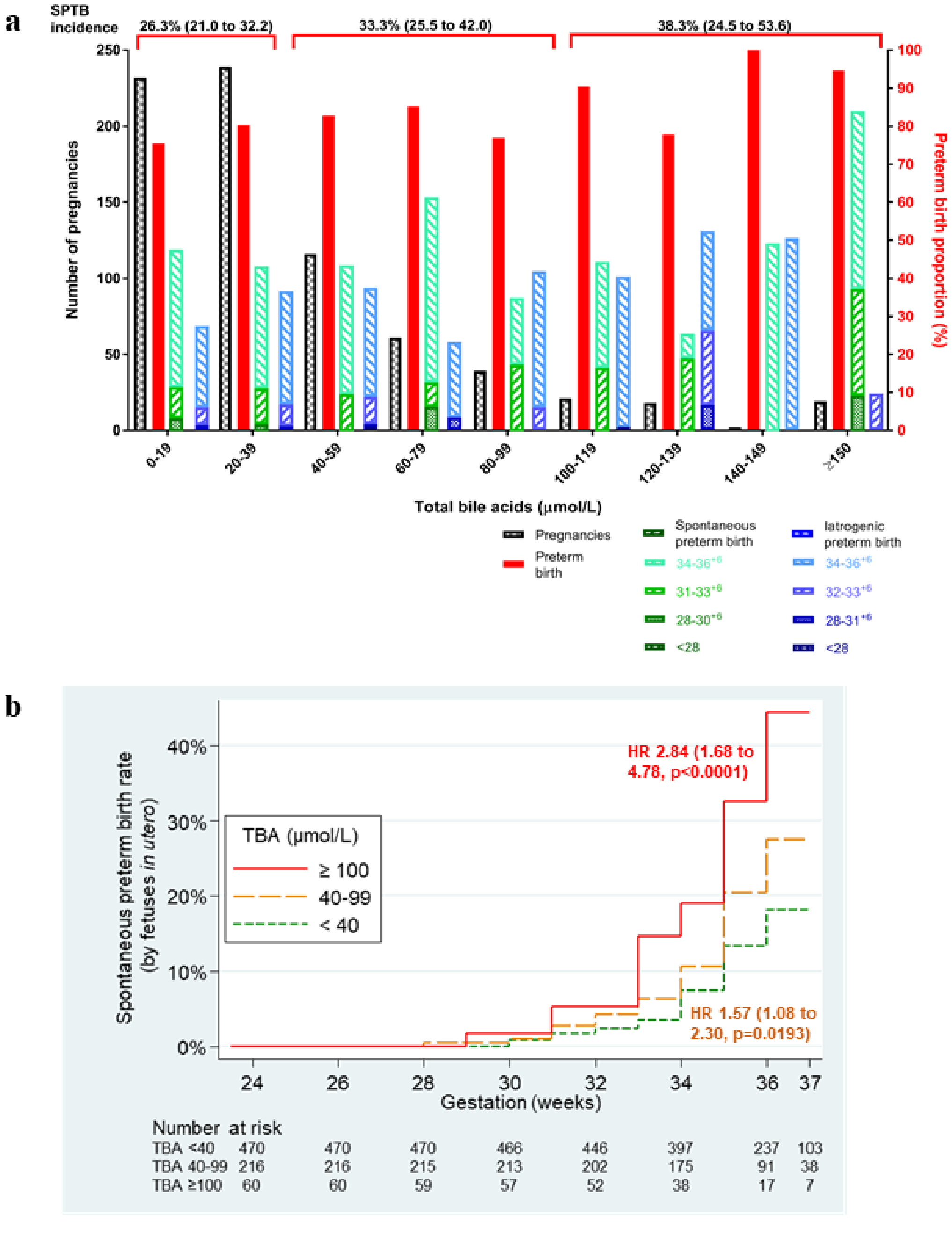

a) Number of women with ICP (black bars) and preterm birth proportion (red bars) by peak total bile acid category. Spontaneous preterm birth proportion: green bars. Iatrogenic preterm birth proportion: blue bars. Spontaneous preterm birth (SPTB) incidence by total bile acid groups <40µmol/L, 40-99µmol/L and ≥100µmol/L (95% confidence interval)

b) Spontaneous preterm birth rate by number of fetuses *in utero* until 37 gestational weeks. Data are analysed by completed gestational week categories, with alterations plotted mid-week to reflect uncertainty by individual day of change. HR: hazard ratio, showing HR of spontaneous preterm birth rate compared with women with bile acids <40µmol/L (95% confidence interval)

References

1 Al Shobaili HA, Hamed HO, Al Robaee A, Alzolibani AA, Amin AF, Ahmad SR. Obstetrical and fetal outcomes of a new management strategy in patients with intra-hepatic cholestasis of pregnancy. *Arch Gynecol Obstet*. 2011; **283**: 1219–1225.

2 Ataalla WM, Ziada DH, Gaber R, Ossman A, Bayomy S, Elemary BR. The impact of total bile acid levels on fetal cardiac function in intrahepatic cholestasis of pregnancy using fetal echocardiography: a tissue Doppler imaging study. *J Matern Fetal Neonatal Med*. 2016; **29**: 1445–1450.

3 Castaño G, Lucangioli S, Sookoian S, et al. Bile acid profiles by capillary electrophoresis in intrahepatic cholestasis of pregnancy. *Clin Sci*. 2006; **110**: 459–465.

4 Cui Y, Xu B, Zhang X, He Y, Shao Y, Ding M. Diagnostic and therapeutic profiles of serum bile acids in women with intrahepatic cholestasis of pregnancy-a pseudo-targeted metabolomics study. *Clin Chim Acta*. 2018; **483**: 135–141.

5 RCOG Green Top Guideline No. 43. Obstetric Cholestasis. 2011 [cited 2018 Mar 17]. Available from: https://www.rcog.org.uk/globalassets/documents/guidelines/gtg_43.pdf

6 Furrer R, Winter K, Schäffer L, Zimmermann R, Burkhardt T, Haslinger C. Postpartum blood loss in women treated for intrahepatic cholestasis of pregnancy. *Obstet Gynecol*. 2016; **128**: 1048–1052.

7 Garcia-Flores J, Cañamares M, Cruceyra M, et al. Clinical value of maternal bile acid quantification in intrahepatic cholestasis of pregnancy as an adverse perinatal outcome predictor. *Gynecol Obstet Invest*. 2015; **79**: 222–228.

8 Geenes V, Chappell LC, Seed PT, Steer PJ, Knight M, Williamson C. Association of severe intrahepatic cholestasis of pregnancy with adverse pregnancy outcomes: A prospective population-based case-control study. *Hepatology*. 2014; **59**: 1482–1491.

9 Glantz A, Marschall HU, Mattsson LÅ. Intrahepatic cholestasis of pregnancy: Relationships between bile acid levels and fetal complication rates. *Hepatology*. 2004; **40**: 467–474.

10 Grymowicz M, Czajkowski K, Smolarczyk R. Pregnancy course in patients with intrahepatic cholestasis of pregnancy treated with very low doses of ursodeoxycholic acid. *Scand J Gastroenterol*. 2016; **51**: 78–85.

11 Heinonen S, Kirkinen P. Pregnancy outcome with intrahepatic cholestasis. *Obstet Gynecol*. 1999; **94**: 189–193.

12 Herrera CA, Manuck TA, Stoddard GJ, et al. Perinatal outcomes associated with intrahepatic cholestasis of pregnancy. ***J Matern Fetal Neonatal Med***. 2018; **31**: 1913–20.

13 Kebapcilar AG, Taner CE, Kebapcilar L, Bozkaya G. High mean platelet volume, low-grade systemic coagulation, and fibrinolytic activation are associated with pre-term delivery and low APGAR score in intrahepatic cholestasis of pregnancy. ***J Matern Fetal Neonatal Med***. 2010; **23**: 1205–1210.

14 Kowalska-Kańka A, Maciejewski T, Niemiec KT. The concentrations of bile acids and erythropoietin in pregnant women with intrahepatic cholestasis and the state of the fetus and newborn. *Med Wieku Rozwoj*. 2013; **17**: 232–245.

15 Liu X, Landon MB, Chen Y, Cheng W. Perinatal outcomes with intrahepatic cholestasis of pregnancy in twin pregnancies. *J Matern Fetal Neonatal Med*. 2016; **29**: 2176–2181.

16 Oztas E, Erkenekli K, Ozler S, et al. Can routine laboratory parameters predict adverse pregnancy outcomes in intrahepatic cholestasis of pregnancy? *J Perinat Med*. 2015; **43**: 667–674.

17 Raz Y, Lavie A, Vered Y, et al. Severe intrahepatic cholestasis of pregnancy is a risk factor for preeclampsia in singleton and twin pregnancies. *Am J Obstet Gynecol*. 2015; **213**: 395.e1-e8.

18 Roncaglia N, Arreghini A, Locatelli A, Bellini P, Andreotti C, Ghidini A. Obstetric cholestasis: outcome with active management. *Eur J Obstet Gynecol Reprod Biol*. 2002; **100**: 167–170.

19 Sargin Oruç A, Seçkin B, Özcan N, Özyer S, Uzunlar Ö, Danişman N. Role of postprandial bile acids in prediction of perinatal outcome in intrahepatic cholestasis of pregnancy. *J Obstet Gynaecol Res*. 2014; **40**: 1883–1889.

20 Shan D, Hu Y, Qiu P, et al. Intrahepatic cholestasis of pregnancy in women with twin pregnancy. *Twin Res Hum Genet*. 2016; **19**: 697–707.

21 Vural Yilmaz Z, Gencosmanoglu Turkmen G, Daglar K, Yılmaz E, Kara O, Uygur D. Elevated red blood cell distribution width is associated with intrahepatic cholestasis of pregnancy. *Ginekol Pol*. 2017; **88**: 75–80.

22 Wikström Shemer EA, Thorsell M, Marschall HU, Kaijser M. Risks of emergency cesarean section and fetal asphyxia after induction of labor in intrahepatic cholestasis of pregnancy: A hospital-based retrospective cohort study. *Sex Reprod Healthc*. 2013; **4**: 17–22.

23 Wong LFA, Shallow H, O'Connell MP. Comparative study on the outcome of obstetric cholestasis. *J Matern Fetal Neonatal Med*. 2008; **21**: 327–330.

24 Zhang Y, Hu L, Cui Y, et al. Roles of PPARγ/NF-κB signaling pathway in the pathogenesis of intrahepatic cholestasis of pregnancy. *PLoS One*. 2014; **9**: 1–11.

25 Bacq Y, le Besco M, Lecuyer AI, et al. Ursodeoxycholic acid therapy in intrahepatic cholestasis of pregnancy: Results in real-world conditions and factors predictive of response to treatment. *Dig Liver Dis*. 2017; **49**: 63–69.

26 Brouwers L, Koster MPH, Page-Christiaens GCML, et al. Intrahepatic cholestasis of pregnancy: Maternal and fetal outcomes associated with elevated bile acid levels. *Am J Obstet Gynecol*. 2015; **212**: 100e1–e7.

27 Chappell LC, Gurung V, Seed PT, Chambers J, Williamson C, Thornton JG. Ursodeoxycholic acid versus placebo, and early term delivery versus expectant management, in women with intrahepatic cholestasis of pregnancy: semifactorial randomised clinical trial. *BMJ*. 2012; **344**: e3799.

28 Chen J, Deng W, Wang J, Shao Y, Ou M, Ding M. Primary bile acids as potential biomarkers for the clinical grading of intrahepatic cholestasis of pregnancy. *Int J Gynecol Obstet*. 2013; **122**: 5–8.

29 Estiú MC, Frailuna MA, Otero C, et al. Relationship between early onset severe intrahepatic cholestasis of pregnancy and higher risk of meconium-stained fluid. *PLoS One*. 2017; **12**: e0176504.

30 Glantz A, Reilly SJ, Benthin L, Lammert F, Mattsson LÅ, Marschall HU. Intrahepatic cholestasis of pregnancy: Amelioration of pruritus by UDCA is associated with decreased progesterone disulphates in urine. *Hepatology*. 2007; **47**: 544–551.

31 Gunaydin B, Bayram M, Altug M, Cevher S, Bozkurt N. Retrospective analysis of maternal,

fetal, and neonatal outcomesof intrahepatic cholestasis of pregnancy at Gazi University. *Turkish J Med Sci*. 2017; **47**: 583–586.

32 Kawakita T, Parikh LI, Ramsey PS, et al. Predictors of adverse neonatal outcomes in intrahepatic cholestasis of pregnancy. *Am J Obstet Gynecol*. 2015; **213**: 570.e1–e8.

33 Kenyon AP, Piercy CN, Girling J, Williamson C, Tribe RM, Shennan AH. Obstetric cholestasis, outcome with active management: a series of 70 cases. *BJOG*. 2002; **109**: 282–288.

34 Kohari K, Ferrara L, Carroll R, Capogna S, Ditchik A, Fox N. Outcome after implementation of a new management strategy for intrahepatic cholestasis of pregnancy. *Am J Obstet Gynecol*. 2015; **212**: S395.

35 Kondrackiene J, Beuers U, Zalinkevicius R, Tauschel HD, Gintautas V, Kupcinskas L. Predictors of premature delivery in patients with intrahepatic cholestasis of pregnancy. *World J Gastroenterol*. 2007; **13**: 6226–6230.

36 Lee RH, Kwok KM, Ingles S, et al. Pregnancy outcomes during an era of aggressive management for intrahepatic cholestasis of pregnancy. *Am J Perinatol*. 2008; **25**: 341–345.

37 Marathe JA, Lim WH, Metz MP, Scheil W, Dekker GA, Hague WM. A retrospective cohort review of intrahepatic cholestasis of pregnancy in a South Australian population. *Eur J Obstet Gynecol Reprod Biol*. 2017; **218**: 33–38.

38 Rook M, Vargas J, Caughey A, Bacchetti P, Rosenthal P, Bull L. Fetal outcomes in pregnancies complicated by intrahepatic cholestasis of pregnancy in a Northern California cohort. *PLoS One*. 2012; **7**: 3–8.

39 Yayla Abide Ç, Vural F, Kılıççı Ç, et al. Can we predict severity of intrahepatic cholestasis of pregnancy using inflammatory markers? *Turkish J Obstet Gynecol*. 2017; **14**: 160–165.

40 Binder T, Salaj P, Zima T, Vítek L. Randomized prospective comparative study of ursodeoxycholic acid and S-adenosyl-L-methionine in the treatment of intrahepatic cholestasis of pregnancy. *J Perinat Med*. 2006; **34**: 383–391.

41 Bolukbas FF, Bolukbas C, Y Balaban H, et al. Intrahepatic cholestasis of pregnancy: spontaneous vs in vitro fertilization. *Euroasian J Hepatogastroenterology*. 2017; **7**: 126–129.

42 Chen H, Zhou Y, Deng D, Hao H, Dang J, Li J. Intrahepatic cholestasis of pregnancy: Biochemical predictors of adverse perinatal outcomes. *J Huazhong Univ Sci Technol Medical Sci*. 2013; **33**: 412–417.

43 Erkenekli K, İskender CT, Onur Topçu H, Ensari T, Uygur D, Danışman N. Are postprandial bile acid levels helpful in predicting perinatal complications in patients with intrahepatic cholestasis of pregnancy? *Cukurova Med J*. 2015; **40**: 212–220.

44 Friberg AK, Zingmark V, Lyndrup J. Early induction of labor in high-risk intrahepatic cholestasis of pregnancy: what are the costs? *Arch Gynecol Obstet*. 2016; **294**: 709–714.

45 Jin J, Pan S, Huang L, Yu Y, Zhong M, Zhang G. Risk factors for adverse fetal outcomes among women with early- versus late-onset intrahepatic cholestasis of pregnancy. *Int J Gynecol Obstet*. 2015; **128**: 236–240.

46 Joutsiniemi T, Timonen S, Linden M, Suvitie P, Ekblad U. Intrahepatic cholestasis of pregnancy: observational study of the treatment with low-dose ursodeoxycholic acid. *BMC Gastroenterol*. 2015; **15**: 92.

47 Labbe C, Delesalle C, Creveuil C, Dreyfus M. Cholestases intrahepatiques gravidiques (CIG) précoces et tardives : étude des complications materno-fœtales. *Gynécologie Obs Fertil Sénologie*. 2018; **46**: 388–394.

48 Lin J, Gu W, Hou Y. Diagnosis and prognosis of early-onset intrahepatic cholestasis of pregnancy: a prospective study. ***J Matern Fetal Neonatal Med***. 2017; 1–7. doi: 10.1080/14767058.2017.1397124

49 Madazli R, Yuksel MA, Oncul M, Tuten A, Guralp O, Aydin B. Pregnancy outcomes and prognostic factors in patients with intrahepatic cholestasis of pregnancy. *J Obstet Gynaecol*. 2015; **35**: 358–361.

50 Oztekin D, Aydal I, Oztekin O, Okcu S, Borekci R, Tinar S. Predicting fetal asphyxia in intrahepatic cholestasis of pregnancy. *Arch Gynecol Obstet*. 2009; **280**: 975–979.

51 Pata O, Vardarelı E, Ozcan A, et al. Intrahepatic cholestasis of pregnancy: correlation of preterm delivery with bile acids. *Turkish J Gastroenterol*. 2011; **22**: 602–605.

52 Riikonen S, Savonius H, Gylling H, Nikkilä K, Tuomi AM, Miettinen TA. Oral guar gum, a gel-forming dietary fiber relieves pruritus in intrahepatic cholestasis of pregnancy. *Acta Obstet Gynecol Scand*. 2000; **79**: 260–264.

53 Zecca E, De Luca D, Barbato G, Marras M, Tiberi E, Romagnoli C. Predicting respiratory distress syndrome in neonates from mothers with intrahepatic cholestasis of pregnancy. *Early Hum Dev*. 2008; **84**: 337–341.

54 Zhang L, Liu X-H, Qi H-B, et al. Ursodeoxycholic acid and S-adenosylmethionine in the treatment of intrahepatic cholestasis of pregnancy: a multi-centered randomized controlled trial. *Eur Rev Med Pharmacol Sci*. 2015; **19**: 3770–3776.

55 Blencowe H, Cousens S, Jassir FB, et al. National, regional, and worldwide estimates of stillbirth rates in 2015, with trends from 2000: a systematic analysis. *Lancet Glob Heal*. 2016; **4**: e98–108.
